# Large increases in methane emissions expected from North America’s largest wetland complex

**DOI:** 10.1126/sciadv.ade1112

**Published:** 2023-03-01

**Authors:** Sheel Bansal, Max Post van der Burg, Rachel R. Fern, John W. Jones, Rachel Lo, Owen P. McKenna, Brian A. Tangen, Zhen Zhang, Robert A. Gleason

**Affiliations:** ^1^ U.S. Geological Survey, Northern Prairie Wildlife Research Center, Jamestown, ND, USA.; ^2^ Texas Parks and Wildlife Department, San Marcos, TX, USA.; ^3^ U.S. Geological Survey, Hydrologic Remote Sensing Branch, Kearneysville, WV, USA.; ^4^ Earth System Science Interdisciplinary Center, University of Maryland, College Park, MD, USA.

## Abstract

Natural methane (CH_4_) emissions from aquatic ecosystems may rise because of human-induced climate warming, although the magnitude of increase is highly uncertain. Using an exceptionally large CH_4_ flux dataset (~19,000 chamber measurements) and remotely sensed information, we modeled plot- and landscape-scale wetland CH_4_ emissions from the Prairie Pothole Region (PPR), North America’s largest wetland complex. Plot-scale CH_4_ emissions were driven by hydrology, temperature, vegetation, and wetland size. Historically, landscape-scale PPR wetland CH_4_ emissions were largely dependent on total wetland extent. However, regardless of future wetland extent, PPR CH_4_ emissions are predicted to increase by two- or threefold by 2100 under moderate or severe warming scenarios, respectively. Our findings suggest that international efforts to decrease atmospheric CH_4_ concentrations should jointly account for anthropogenic and natural emissions to maintain climate mitigation targets to the end of the century.

## Teaser

Future increases in wetland CH_4_ emissions due to climate warming may offset anthropogenic CH_4_-reduction actions.

## INTRODUCTION

Atmospheric concentrations of methane (CH_4_) have increased over 150% from preindustrial levels and are growing twice as fast as carbon dioxide (CO_2_) (*1*, *2*). Methane’s high radiative efficiency (i.e., ability to absorb heat) makes it several times stronger than CO_2_ in its ability to warm the climate, but it also has a relatively short lifetime (~10 years) in the atmosphere (*3*). These properties make reducing CH_4_ emissions a tenable opportunity to slow the pace of climate change (*4*, *5*). There is widespread international support to cut anthropogenic CH_4_ emissions, as demonstrated through the Global Climate Pledge at the 2021 United Nations Climate Change Conference (COP26) (*6*). Anthropogenic CH_4_ emissions come from quantifiable and manageable sources in the energy, agriculture, and waste sectors (*7*). However, aquatic ecosystems such as wetlands naturally produce CH_4_ in their anoxic soils and collectively account for up to half of total global CH_4_ emissions (*8*). Wetland CH_4_ emissions are sensitive to temperature and will likely increase in response to human-induced climate warming (*2*, *9*–*11*). Consequently, the increase in natural CH_4_ emissions from wetlands may offset progress made from anthropogenic CH_4_-reduction actions, effectively maintaining or elevating atmospheric CH_4_ concentrations to the end of the century. Despite the important role of wetlands in global CH_4_ budgets, there are large discrepancies among global models in terms of CH_4_ emissions estimates, particularly at regional scales (*12*). These discrepancies add to the uncertainty surrounding current and future global CH_4_ emissions estimates. Improved regional wetland CH_4_ models are needed to inform and constrain the global models that are used to develop climate change mitigation policies.

Uncertainty in estimates of wetland CH_4_ emissions is driven, in part, by differences in modeling approaches (e.g., top-down versus bottom-up), model complexity and forcing data, and spatiotemporal prediction resolution. Furthermore, using coarse-scale models to infer fine-scale processes may also perpetuate biases and errors when upscaling to larger areas, referred to as “the ecological inference fallacy” (*13*). For instance, global models predict wetland CH_4_ flux over large grid cells (e.g., 0.5 arc degrees or larger) (*2*, *14*). However, the processes that govern wetland CH_4_ fluxes occur over very fine spatial scales (meters) and may not scale linearly with average conditions or the inundated fractions of large grid cells.

**Fig. 1.**
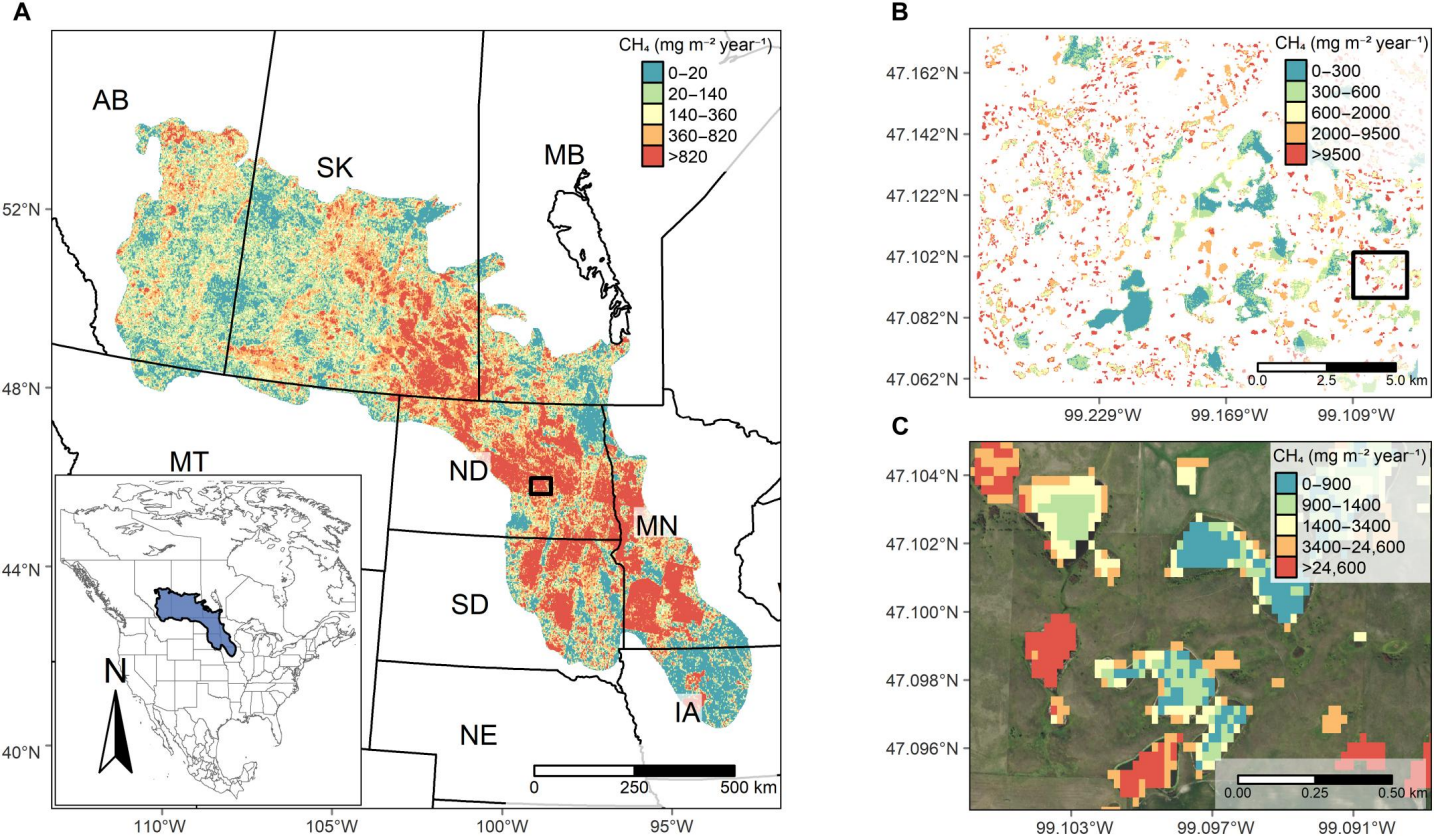
Spatially explicit model results of wetland CH_4_ emissions from the PPR in 2011. (**A**) Ecosystem-scale map of wetland CH_4_ emissions (mg m^−2^ year^−1^) from the PPR in central North America (inset map) in 2011, representing a wet period in the region. (**B**) Map of wetland CH_4_ emissions demonstrating a high level of among-wetland variation. (**C**) Fine-resolution (30-m pixel) map showing within-wetland variation in CH_4_ emissions. Boxes in maps (A) and (B) are areas used for maps (B) and (C), respectively. Maps (B) and (C) represent locations with relatively high wetland densities. Legend colors correspond with quantiles of CH_4_ flux rates within each map to help visualize spatial variation in CH_4_ fluxes across the region in (A), among wetlands in (B), and within wetlands in (C). Base map used in (A) from rnaturalearth: World Map Data from Natural Earth (https://docs.ropensci.org/rnaturalearth). Background aerial image in (C) from National Agriculture Imagery Program (NAIP) Digital Ortho Photo Image (www.fisheries.noaa.gov/inport/item/49508). AB, Alberta; SK, Saskatchewan; MB, Manitoba; MT, Montana; ND, North Dakota; SD, South Dakota; MN, Minnesota; IA, Iowa; NE, Nebraska.

Creating fine-resolution models of CH_4_ emissions across the landscape requires spatially extensive CH_4_ flux measurements with covarying environmental input data. We address these data and modeling issues using a one-of-a-kind CH_4_ dataset with nearly 19,000 chamber flux measurements from wetlands in the Prairie Pothole Region (PPR) of North America, the largest wetland complex on the continent (~820,000 km^2^) and 10th largest in the world (*15*). These CH_4_ flux measurements were collected from 143 wetlands nested within both native prairie and agricultural fields distributed across >200,000 km^2^ over 13 years. We use these flux data along with field-measured or remotely sensed environmental variables to develop a chamber (plot-scale) model to better understand the key drivers of CH_4_ flux. We then use the chamber model to inform a spatially explicit landscape-scale model of CH_4_ emissions from PPR wetlands (Fig. 1, Materials and Methods, and the Supplementary Materials). We use the landscape model to (i) estimate the historical range of natural variation in PPR wetland CH_4_ emissions, (ii) compare against bottom-up and top-down global wetland CH_4_ emissions models, and (iii) predict future wetland CH_4_ emissions under four different climate scenarios in the PPR.

PPR wetlands are mineral soil, shallow depressions carved into the landscape by late Pleistocene glaciers. PPR wetlands span a wide range of sizes (<0.01 to >10 ha), hydroperiods (ephemeral to permanent), vegetation (open waters to dense macrophytes), and salinities (fresh to hypersaline) (*16*, *17*). Less than 0.2% of depressions in the PPR resemble lakes because of their large surface areas (e.g., >1 km^2^), although they have relatively shallow depths (<2 m) and are thus considered wetlands here. Wetlands of the PPR are heavily affected by agricultural practices such as drainage, tillage, and fertilizer application. PPR wetlands are also highly valued by society because they provision crucial habitat for migratory waterfowl in North America (*18*), serve as biochemical reactors that recycle nutrients (*19*), and support long-term sequestration and storage of atmospheric carbon (*20*). Some of the highest CH_4_ emissions have been reported from PPR wetlands (*21*–*25*), which suggests that the region may contribute substantially to North American CH_4_ budgets.

## RESULTS AND DISCUSSION

### Chamber (plot-scale) wetland methane flux: The role of hydrology, temperature, vegetation, and land use

**Fig. 2.**
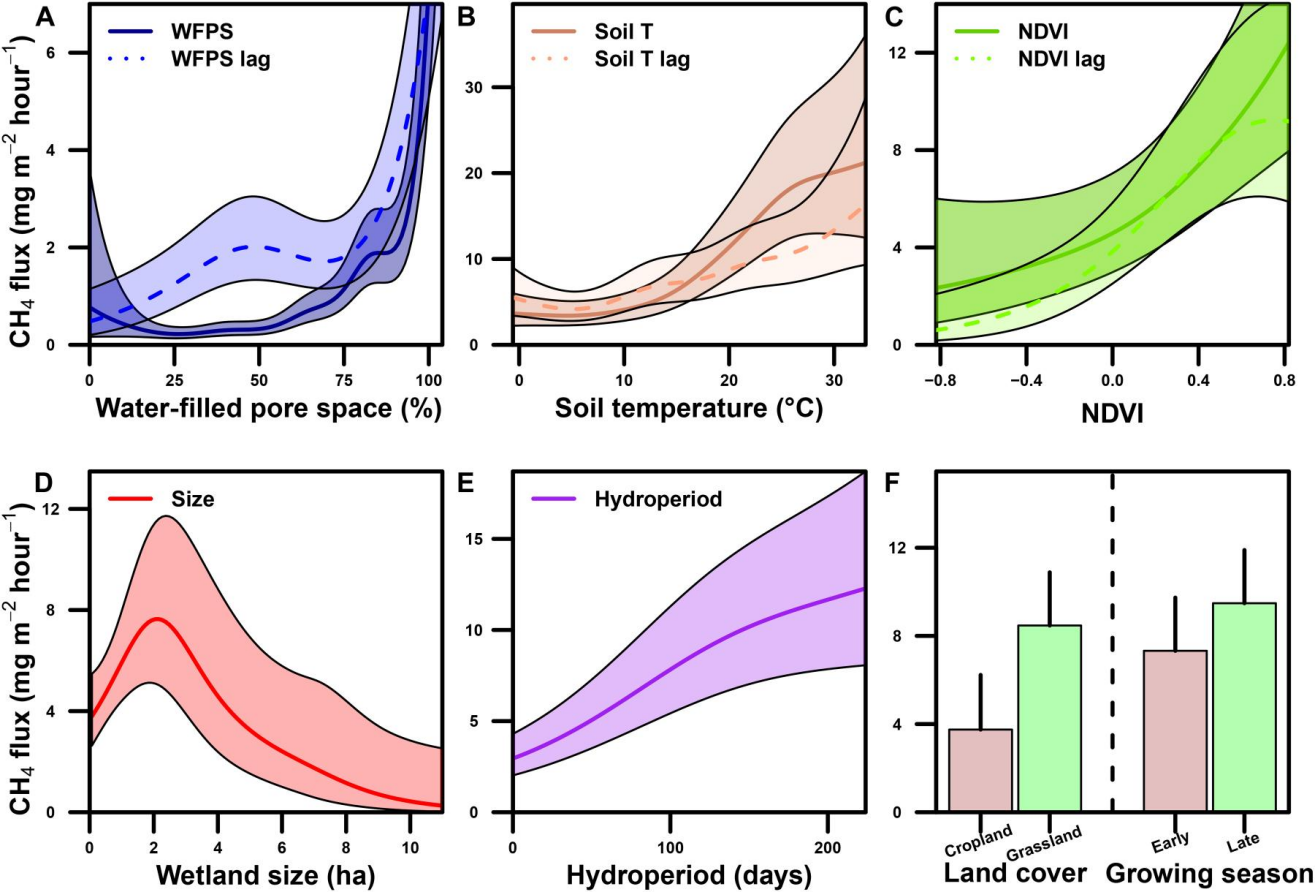
Graphical depiction of functional relationships between CH_4_ flux rates (mg m^−2^hour^−1^) and explanatory variables in PPR wetlands using generalized additive modeling. Relation between CH_4_ flux and (**A**) WFPS and its lag (WFPS lag)* (%), (**B**) soil temperature (Soil T) and its lag (Soil T lag)* (degrees Celsius), (**C**) NDVI and its lag (NDVI lag)*, (**D**) wetland size (hectares), (**E**) hydroperiod (days), and (**F**) (left) surrounding land cover and (right) growing season interval [i.e., first (early) or second (late) half of the growing season]. *Lag predictors are first-order lags from the prior 2-week time step. Solid lines and dashed lines in (A) to (E) and bars in (F) represent mean estimates. Shaded regions in (A) to (E) and vertical lines on bars of (F) represent 95% confidence intervals.

Our exceptionally large flux dataset provides a unique opportunity to disentangle the key drivers of CH_4_ flux that are relevant to mineral soil wetlands in temperate regions, particularly those that are depressional and affected by agriculture (e.g., throughout the Great Plains of North America and kettle holes in Northern Europe). We developed our chamber model using generalized additive models to capture nonlinear responses of CH_4_ flux to explanatory environmental variables (Fig. 2). We identified (i) water-filled pore space (WFPS), (ii) soil temperature, (iii) normalized difference vegetation index (NDVI; a measure of plant activity and biomass), (iv) wetland size, (v) hydroperiod, (vi) surrounding land cover, and (vii) growing season interval (i.e., first or second half of the growing season) as important variables explaining CH_4_ fluxes (63% deviance explained, Fig. 2).

Each of the explanatory variables in our chamber model has been identified in other studies as mechanistic drivers of CH_4_ flux from wetlands (additional description on chamber model variables in Materials and Methods and the Supplementary Materials) (*9*, *26*–*28*). Soil saturation, temperature, and vegetation are all fundamentally linked to wetland CH_4_ emissions (*9*, *29*). We found that flux rates of CH_4_ sharply declined when surface soil saturation (top 5 cm) fell below 80% WFPS (Fig. 2A). Studies have shown that even a thin oxic layer can rapidly stimulate CH_4_ oxidation and limit CH_4_ emissions by up to 90% (*30*, *31*). The nonlinear increase in CH_4_ fluxes at higher temperatures roughly followed an Arrhenius metabolic-response pattern, which was likely caused by a direct stimulation of methanogenic CH_4_ production in wetland soils (Fig. 2B; see table S1 for *Q*_10_ coefficients) (*9*, *10*). The strong temperature sensitivity of CH_4_ production is a key parameter driving seasonal and interannual variation in wetland CH_4_ models (*11*, *32*).

The steep increase in CH_4_ flux when NDVI values transitioned from negative to positive likely represents the large contribution of emergent vegetation to CH_4_ emissions (Fig. 2C) (*33*, *34*). Elevated CH_4_ emissions have been mechanistically linked to the invasive *Typha* × *glauca* (hybrid cattail). Hybrid cattail, like other emergent species, adds fresh carbon substrates to soils that fuel methanogenesis and provides a transport pathway through its stems that facilitates CH_4_ emissions (*35*). This invasive taxa of cattail is prevalent in wetlands of the PPR and is spreading throughout much of North America (*36*, *37*), potentially increasing the CH_4_ footprint of the landscape. We also found that first-order lags of WFPS, soil temperature, and NDVI were important in explaining CH_4_ fluxes, which indicates that antecedent (~2 weeks earlier) conditions are important in predicting CH_4_ production and emissions. Understanding the functional relationships between explanatory variables and CH_4_ emissions is an important step in the development of low-complexity models.

Although many PPR wetlands have been drained, the vast majority of remaining, undrained wetlands are nested within agricultural fields, which can affect greenhouse gas fluxes (*22*). Notably, after accounting for other explanatory variables, CH_4_ fluxes were marginally greater from wetlands surrounded by grasslands compared to those nested in croplands (Fig. 2F). This result was contradictory to the assumption that fertilizer inputs from agricultural runoff increase aquatic CH_4_ emissions (*38*). In the PPR, wetlands that are nested within croplands not only may experience nutrient enrichment (*18*) but also have less soil organic carbon substrates to fuel methanogenesis (*25*, *38*, *39*) and more aerated soils to sustain CH_4_ oxidation. These factors may explain why we found lower CH_4_ emissions from wetlands nested in croplands compared to grasslands. Future projections of land use show disparate futures with cropland cover either increasing or decreasing (*40*). Regardless, the overall effect of surrounding landcover on CH_4_ flux was relatively weak (e.g., explained 2% of variation in the landscape model; fig. S1), possibly because we only considered two generic types of landcover (i.e., “grassland” and “cropland”) to facilitate upscaling. Future modeling efforts should consider including more detailed information on land use, such as crop species, fertilizer inputs, and tillage intensity, to improve estimates of how upland management in the PPR affects wetland greenhouse gas emissions.

### Chamber (plot-scale) wetland methane flux: The role of wetland size

**Fig. 3.**
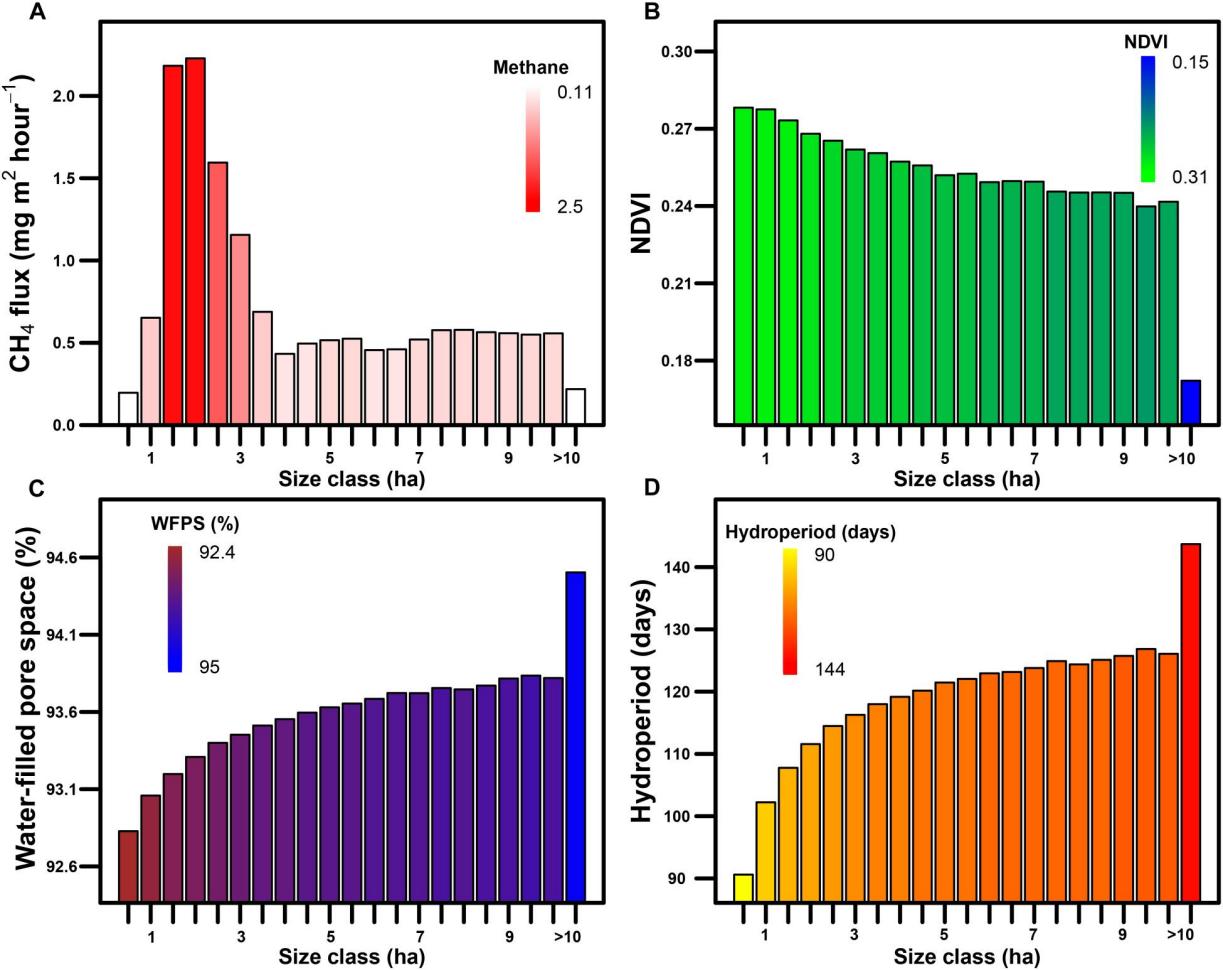
Changes in wetland CH_4_ flux rates (mg m^−2^hour^−1^) and predictor variables in 0.5-hectare (ha) size classes for the PPR of North America in 2011. (**A**) Mean wetland CH_4_ flux rates, (**B**) NDVI values, (**C**) WFPS, and (**D**) hydroperiod in 0.5-ha size classes. The largest size class (>10 ha) includes wetlands that resemble lakes because of their surface areas, although most are also shallow (<2 m) and considered wetlands in this study.

Wetland or lake size has repeatedly been identified as an important predictor of aquatic CH_4_ flux, with exponential increases in CH_4_ flux rates with decreasing size (*26*, *41*, *42*). Our model shows a similar relationship at the larger end of the size gradient, with large wetlands having low CH_4_ flux rates relative to their surface area (Fig. 2D). At the smaller end of the size gradient, CH_4_ flux rates sharply increased and peaked in medium-sized wetlands around 2 to 4 ha in size, while the very smallest wetlands less than 1 ha had notably lower flux rates (Fig. 3A). The convex relationship that we found between CH_4_ flux rates and size contradicts the studies that show unconstrained increases in CH_4_ emissions as waterbody size decreases (e.g., in ponds and lakes) (*26*). There are a number of physical and biogeochemical factors that cause the differences in CH_4_ emissions among small (<1 ha)-, medium (2 to 4 ha)-, and large (>10 ha)-sized wetlands. For example, the smallest wetlands have drier soils and shorter hydroperiods than larger wetlands (Fig. 3, C and D), shortening the period of CH_4_ production and increasing the potential for CH_4_ oxidation (*35*). Large wetlands tend to have relatively high salinity, lower soil carbon content, and less vegetation (*43*), which helps explain their low CH_4_ flux rates per unit area. Medium-sized wetlands have relatively wet soils and long hydroperiods compared to the smallest wetlands and can also support more macrophytic vegetation such as hybrid cattail compared to larger wetlands (Fig. 3, B to D) (*44*). Thus, medium-sized wetlands are in the “goldilocks” zone, meaning that conditions are especially favorable for CH_4_ production and emissions.

### Landscape wetland methane emissions: Historical dry versus wet periods

Climate and land management both have the potential to alter wetland hydrology and, thus, the total extent of surface water (referred to here as “wetland extent”) in the PPR. Understanding the impacts of wetland extent on CH_4_ emissions is important because wetland hydrology may offset or magnify the effects of climate warming on future emissions. Unlike many parts of the world that are experiencing drought, the PPR underwent a marked increase in wetland extent beginning in the 1990s after a 4-year drought (Table 1) (*45*). To understand the implications of this expansion in wetland extent, we estimated cumulative wetland CH_4_ emissions across the PPR for the years 1991 and 2011 (*46*). The year 1991 was the driest in a multiyear drought (1988 to 1992), where wetland surface water levels were at historic lows. Conversely, 2011 was an unusually wet year with ~20 antecedent wet years that represented the largest recorded extent of wetlands (*45*). These 2 years represent “bookends” of potential wetland extent in the PPR (Materials and Methods).

**Table 1.** Mean wetland CH_4_ emissions (gigagrams), flux rates (mg m^−2^hour^−1^), and environmental predictors by location and historical climate condition or future climate scenarios for the PPR of North America. CAN, Canada; PPR, Prairie Pothole Region*; SSP, socioeconomic pathway; Soil T, soil temperature. *PPR aggregates data from the United States and Canada. For CH_4_ flux rates, the values in parentheses represent the coefficient of variation (CV; expressed as a percent) around the prediction means of 13 Earth system models (ESMs) from the Coupled Model Intercomparison Project Phase 6 (CMIP6). For environmental predictors, values in parentheses represent the CV of values used in the models. Note that values from historical conditions do not have CV values because they were not based on multiple models.

Country	Year	Scenario	Condition	Annual CH_4_ emission (Gg)	CH_4_ flux rate (mg m^−2^ hour^−1^)	Growing season (days)	Inundated area (km^2^)	Wetland count (×1000)	NDVI	Soil T (°C)	Hydroperiod (days)
CAN	1991	Historical	Dry	172.9	0.57	266	22,941	2570	0.19	11.4	120
CAN	2011	Historical	Wet	259.9	0.85	210	34,431	2947	0.22	12.0	126
CAN	2100	SSP2-4.5	Dry	390.3 (26)	1.27 (25)	280 (7)	22,934	2570	0.16 (8)	13.4 (6)	134 (6)
CAN	2100	SSP2-4.5	Wet	618.5 (23)	1.91 (22)	280 (7)	34,419	2945	0.17 (7)	13.4 (6)	158 (6)
CAN	2100	SSP5-8.5	Dry	602.3 (16)	1.77 (10)	321 (9)	22,934	2570	0.13 (18)	14.4 (7)	157 (11)
CAN	2100	SSP5-8.5	Wet	909.5 (15)	2.57 (10)	321 (9)	34,419	2945	0.14 (17)	14.4 (7)	183 (10)
USA	1991	Historical	Dry	178.9	1.33	266	8,890	1209	0.20	13.5	137
USA	2011	Historical	Wet	360.8	1.67	280	21,794	1846	0.20	13.4	145
USA	2100	SSP2-4.5	Dry	320.1 (10)	2.24 (8)	323 (6)	8,889	1209	0.17 (6)	14.9 (4)	162 (7)
USA	2100	SSP2-4.5	Wet	652.4 (9)	2.63 (7)	323 (6)	21,793	1846	0.16 (10)	15.0 (4)	176 (6)
USA	2100	SSP5-8.5	Dry	369.3 (8)	2.41 (6)	337 (5)	8,889	1209	0.16 (8)	16.3 (7)	180 (8)
USA	2100	SSP5-8.5	Wet	760.1 (9)	2.84 (5)	337 (5)	21,793	1846	0.14 (12)	16.4 (7)	195 (9)
PPR	1991	Historical	Dry	351.8	0.85	266	31,832	3779	0.19	12.2	126
PPR	2011	Historical	Wet	620.7	1.16	280	56,225	4793	0.21	12.5	133
PPR	2100	SSP2-4.5	Dry	710.4 (19)	1.63 (16)	323 (6)	31,823	3779	0.17 (7)	13.9 (5)	144 (6)
PPR	2100	SSP2-4.5	Wet	1270.9 (16)	2.18 (15)	323 (6)	56,212	4793	0.16 (8)	14.0 (5)	165 (6)
PPR	2100	SSP5-8.5	Dry	971.5 (13)	2.01 (8)	337 (5)	31,823	3779	0.14 (13)	15.1 (7)	165 (10)
PPR	2100	SSP5-8.5	Wet	1669.6 (12)	2.67 (8)	337 (5)	56,212	4793	0.14 (15)	15.1 (7)	187 (9)

We fit our landscape model using a random forest (RF) machine learning (ML) algorithm. We used the functional relationships from the chamber model (Fig. 2) to develop and tune our RF model (fig. S1; additional description of the landscape model in Materials and Methods and the Supplementary Materials). For each variable in our chamber model, we acquired a remotely sensed surrogate variable at a 30-m resolution. For example, variables associated with the presence, extent, and permanence of water (i.e., WFPS, size, and hydroperiod, respectively) were generated using the dynamic surface water extent (DSWE) algorithm. DSWE has proven effective at identifying both open water and vegetated wetland pixels in the PPR and other wetland ecosystems (*47*). It should be noted that we assumed low CH_4_ emissions in winter based on negligible flux rates that we measured over frozen soils, snow, or ice (see Materials and Methods), which may have underestimated cumulative annual CH_4_ emissions. Processes that occur during freeze-up in autumn, under ice during winter, or thaw in spring have been identified as potential “hot moments” of substantial CH_4_ emissions (*48*–*51*).

**Fig. 4.**
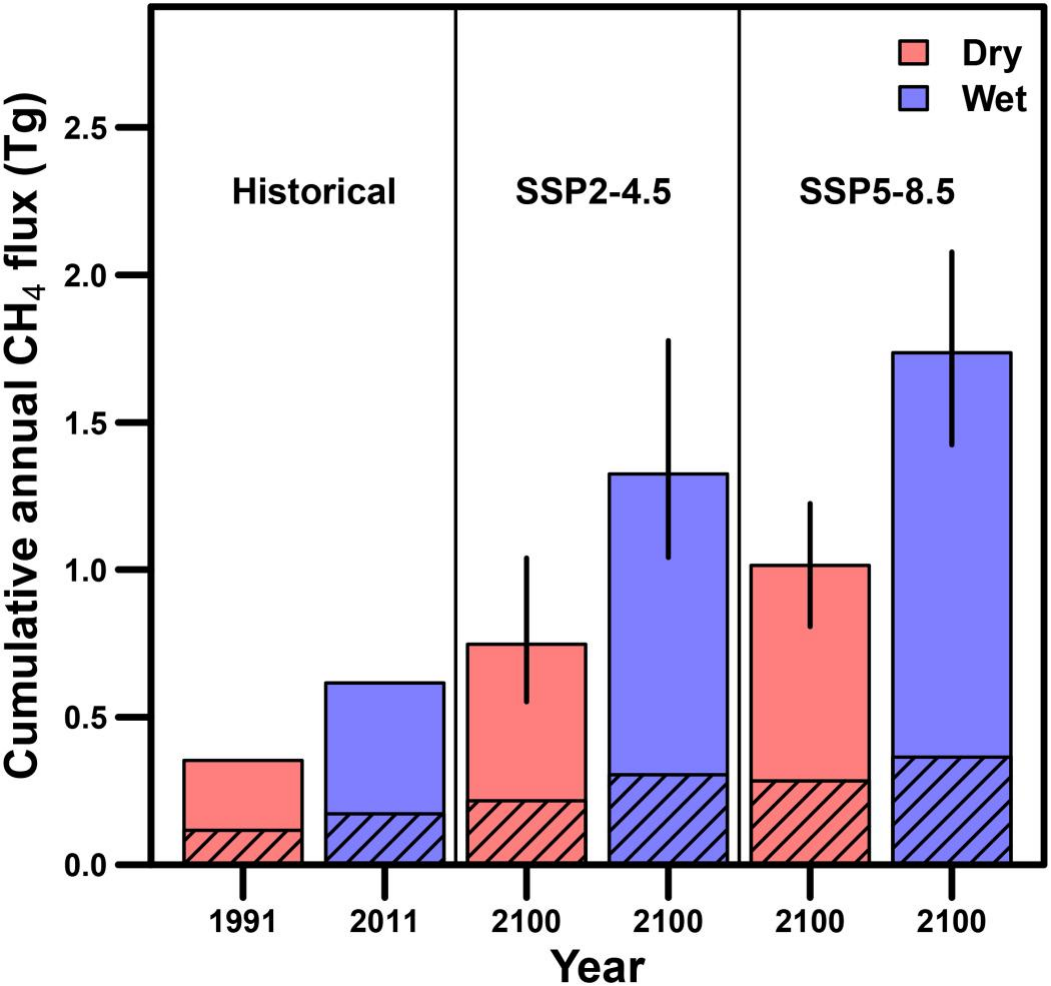
Cumulative wetland annual CH_4_ emissions (teragrams) from the PPR under two historical climate conditions and four future climate scenarios. Historical conditions include 1991 and 2011 (left two bars), representing historical dry (red) and wet (blue) years, respectively. Future climate scenarios include two socioeconomic pathways (SSPs) based on 13 Earth system models (ESMs) from the Coupled Model Intercomparison Project Phase 6 (CMIP6). SSP2-4.5 (middle two bars) and SSP5-8.5 (right two bars) represent moderate (~1.7°C) and severe (~2.7°C) warming scenarios, respectively. Both SSPs were run under potential dry or wet future hydrologic conditions due to high uncertainty in future wetland extent. Hatched and unhatched portions of each bar represent larger (>10 ha) and smaller (<10 ha) wetland contributions to CH_4_ emissions, respectively. Error bars for future scenarios represent the range of predictions across the 13 ESMs.

**Fig. 5.**
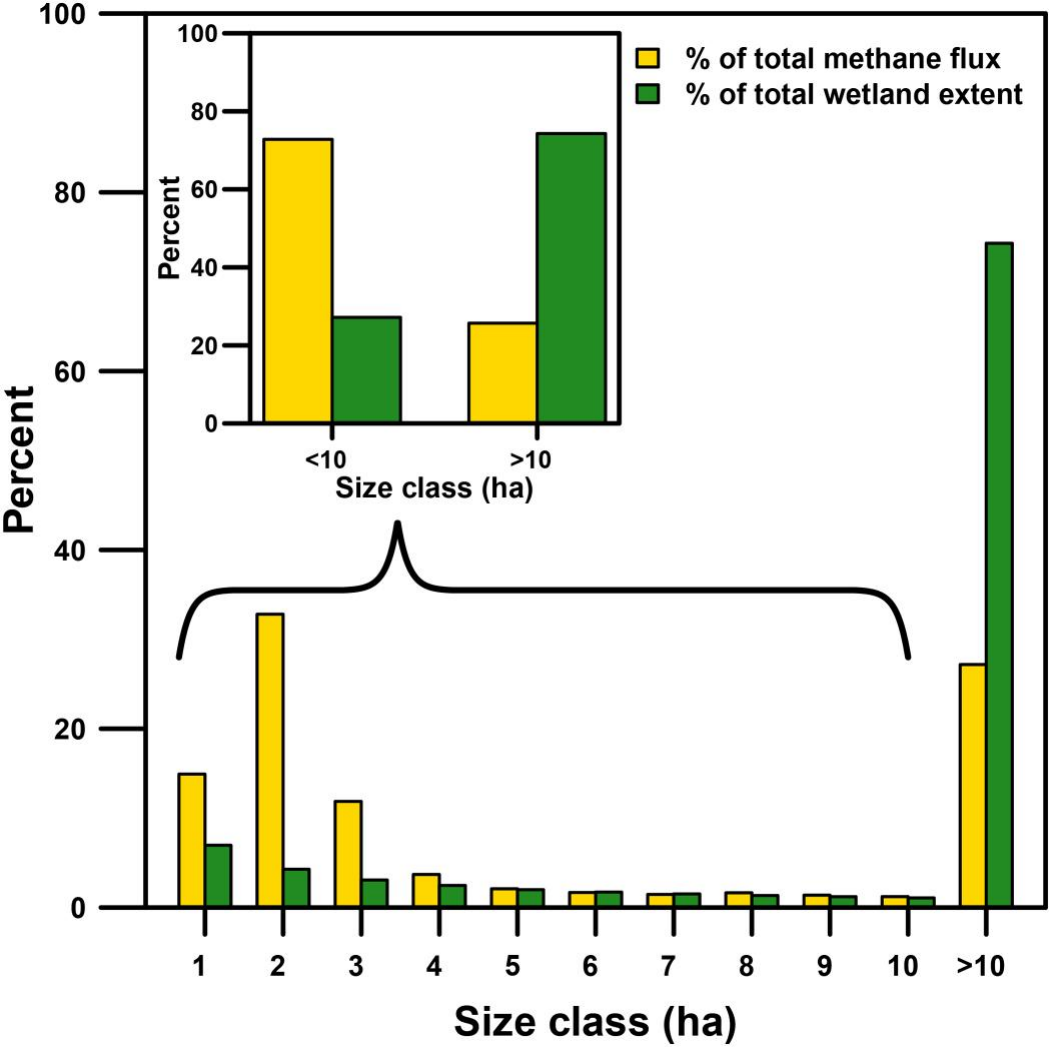
The relative percent (%) of wetland CH_4_ flux (yellow) and percent of wetland extent (green) across the PPR in 2011 by 1 ha wetland size classes up to 10 ha and greater. The inset graph aggregates percent CH_4_ flux and percent wetland extent for wetlands less than or greater than 10 ha (0.1 km^2^). Wetland less than 10 ha contribute ~75% of CH_4_ emissions but only represents ~25% of total wetland surface extent.

We classified 3.8 million wetlands in 1991 and 4.8 million in 2011 using the DSWE algorithm at a 30-m resolution. Across the PPR, wetland extent nearly doubled from ~31,000 to ~56,000 km^2^ between 1991 and 2011. This doubling of wetland extent was the primary driver of cumulative, annual CH_4_ emissions increasing nearly twofold from dry to wet conditions (from 0.35 to 0.62 Tg of CH_4_ emissions, respectively; mean, ~0.5 Tg of CH_4_ year^−1^; Fig. 4, fig. S2, and Table 1). These annual emission estimates are, contrary to our initial expectations, relatively low when compared to global wetland (~101 to 179 Tg of CH_4_ year^−1^ for 2000 to 2017) or anthropogenic CH_4_ emissions (*52*). PPR wetland CH_4_ emissions in 2011 (0.62 Tg of CH_4_) equated to about 2% of anthropogenic emissions from Canada and the United States (combined total, ~35 Tg of CH_4_ in 2010; https://cfpub.epa.gov/ghgdata/nonco2/). Low CH_4_ emissions from the PPR are attributable, in part, to the distribution of wetland sizes and to the convex relationship between CH_4_ flux and wetland size. Specifically, wetlands larger than 10 ha make up the majority of the inundated area but contribute a relatively small fraction of total CH_4_ emissions (Fig. 5).

### Landscape wetland methane emissions: Comparisons to global models

We next considered how our estimates of total CH_4_ emissions compared to individual and ensemble global models. We made comparisons with 13 biogeochemical, bottom-up models and 21 top-down atmospheric inversion models used in the Global Carbon Project version 2 (GCPv2) and the 18-member bottom-up models of Wetland Methane Emissions and Uncertainty (WetCHARTs) v1.3 (*2*, *14*). Over the same year (2011) and region (PPR), ensemble estimates of annual CH_4_ emissions were similar to our estimate. Even so, individual model estimates were 1.5- to 5-fold lower or higher than our estimate (table S2). The discrepancies among models are caused, in part, by shifts in scale between the different models; e.g., our fine-scale model of wetland CH_4_ flux provides additional information missed by coarser, bottom-up and top-down models that predict over 0.5° or larger grid cells (fig. S3). Our fine spatial resolution model was able to capture among- and within-wetland variation in CH_4_ flux rates (i.e., not all wet pixels in a 0.5° grid cell are equal; Fig. 1, B and C). The significance of considering fine-scale spatial variability can be seen when considering that wetlands less than 10 ha in size accounted for only ~25% of total wetland extent but contributed ~75% to total annual CH_4_ emissions (Fig. 5). This finding demonstrates why scaling to the fraction of inundated area in large grid cells can miss important fine-scale spatial processes and can misrepresent landscape-scale CH_4_ emissions. These findings have large implications for global models because they now do not include wetland size as a predictor due, in part, to inadequate global wetland mapping products.

Despite differences in annual wetland CH_4_ emission estimates between our PPR model and other individual global models, our estimate of 0.62 Tg of CH_4_ for 2011 was relatively close to ensemble CH_4_ estimates of both WetCHARTs v1.3.1 (median, ~0.66 Tg of CH_4_) and GCPv2 bottom-up models (mean, ~0.58 Tg of CH_4_) (table S2 and Materials and Methods). In addition, predicted CH_4_ emissions among bottom-up, top-down, and our PPR models followed similar spatial patterns across the landscape as seen in fig. S3. The CH_4_ estimates between our PPR model and the GCPv2 bottom-up models were closer when GCPv2 models used the Wetland Area and Dynamics for Methane Modeling (WAD2M) wetland maps (table S2) (*2*, *53*). WAD2M maps account for dynamic seasonality of wetland inundation, which is similar to the methodology that we used to characterize inundation. Both WAD2M and our PPR model estimated similar wetland extents in the PPR for 2011 (50,229 versus 56,225 km^2^, respectively). These results suggest that aggregated ensembles of process-based, bottom-up models can reduce bias, especially when using seasonally dynamic wetland extents. Even so, both top-down and bottom-up models still need more spatial validation using independent estimates like our regional PPR CH_4_ model to (i) inform and evaluate individual models, (ii) optimize ensembles of bottom-up models, and (iii) provide priors to inform top-down inversion models, all of which will lead to more accurate estimates and partitioning of global CH_4_ emissions from wetlands and other sources.

### Landscape wetland methane emissions: Future climate scenarios

We next used our landscape model to estimate cumulative wetland CH_4_ fluxes using future temperatures derived from 13 Earth system models (ESM) associated with socioeconomic pathway (SSP) 2-4.5 and SSP5-8.5 [corresponding to representative concentration pathway 4.5 (RCP4.5) and RCP8.5]. SSP2-4.5 and SSP5-8.5 represent “middle of the road” (moderate warming, ~1.7°C PPR) and “intensive fossil fueled” (severe warming, ~2.7°C PPR) emission trajectories, respectively (*54*). While ESMs have relatively high confidence and agreement on future temperature predictions, there is much greater uncertainty and disagreement of future precipitation patterns among ESMs in the PPR and elsewhere (*55*). Moreover, policy and land management decisions in both the United States and Canada will influence the relative amounts of wetland drainage, conservation, and restoration (*56*). Such decisions, along with changing climate drivers, could decrease, maintain, or increase wetland extent. Because of high uncertainty in future inundation, we decided to use 1991 and 2011 wetland extents as “dry” and “wet” bookends, respectively, to capture the potential range of future cumulative CH_4_ emissions. We modeled four climate scenarios: (i) moderate warming and dry, (ii) moderate warming and wet, (iii) severe warming and dry, and (iv) severe warming and wet.

Our model estimates show that cumulative annual CH_4_ emissions are expected to increase ~2-fold under the SSP2-4.5 (mean, 0.99 Tg of CH_4_) and ~3-fold under the SSP5-8.5 (mean, 1.32 Tg of CH_4_) scenarios (overall mean, 1.16 Tg of CH_4_ in 2100; Fig. 4 and Table 1). The predicted increase in future PPR CH_4_ emissions is attributable, in part, to the high sensitivity of CH_4_ flux rates to increasing soil temperatures and to longer (~50 more days) frost-free growing seasons (Table 1, table S3, Fig. 2, and fig. S1). Temperature and growing season length are both consistently strong drivers of annual CH_4_ emissions across wetland types (*9*, *28*, *50*). Total CH_4_ emissions vary considerably among ESMs, with differences of ~0.5 (0.42 to 0.73) Tg of CH_4_ within each scenario or, approximately, 20 to 40% variation around the means (Fig. 4). The contribution from larger wetlands (>10 ha) ranged from ~20 to 30% of total CH_4_ emissions. Within SSP2-4.5 and SSP5-8.5, the magnitudes of predicted increases in annual CH_4_ emissions are strongly dependent on future wetland extent, with nearly twice the CH_4_ emissions if conditions are wet compared to dry. However, even if conditions in the future are relatively dry, moderate or severe warming is predicted to increase future landscape-scale wetland CH_4_ emissions. Future wetland CH_4_ emissions will be highly dependent on CH_4_ flux rates per unit area, growing season length, and total wetland extent.

Annual CH_4_ emission estimates from PPR wetlands are ~0.5 Tg of CH_4_ year^−1^, and future emissions to the end of the century are projected to be ~1.2 Tg of CH_4_ year^−1^. These estimates seem low compared to global studies that infer total temperate wetland emissions of 25 ± 11 Tg of CH_4_ year^−1^ (*2*). However, despite being the largest wetland complex in North America, PPR wetlands only accounts for 10 to 15% of mineral soil wetland in North America and under 2% of temperate wetlands globally (*53*, *57*). If we assume that other temperate, mineral soil wetlands respond to climate warming similarly as PPR wetlands, then their collective future CH_4_ emissions may substantially exceed present-day emissions. These “excess” CH_4_ emissions from wetlands can arguably be attributed to human-driven climate change. Now, the Intergovernmental Panel on Climate Change (IPCC) guidelines state that CH_4_ emissions from mineral soil wetlands only contribute toward national greenhouse gas inventories if wetland waters are altered by human actions, i.e., drained, rewetted, or created by flooding (*58*). Whereas changes in natural CH_4_ emissions due to human-induced climate warming are not accounted for as anthropogenic sources. Regardless of attribution, the potential future increase in wetland CH_4_ emissions has important implications for climate mitigation strategies.

Reduction of atmospheric CH_4_ will require implementing a wide range of mitigation actions, many of which are expensive, rely on underdeveloped technologies, and/or need considerably long delivery times (*5*, *59*). Less than 10% of all anthropogenic CH_4_ emissions are reducible at low cost, followed by exponentially increasing abatement costs for additional cuts to CH_4_ (*59*). Moreover, over 70% of anthropogenic CH_4_ emissions are now beyond what available technologies can mitigate (*59*). As a result, using natural systems to offset greenhouse gas emissions, the so-called nature-based climate solutions, is gaining interest by national governments. Wetland management in the PPR historically involved disproportionately draining the smallest (low-CH_4_–emitting) wetlands and consolidating them into fewer, larger wetlands (*60*). The resultant changes in wetland size distribution directly, albeit unintentionally, affect landscape-scale CH_4_ emissions. Future strategic management of the wetlandscape such as protection of the smallest (low-CH_4_–emitting) wetlands could lower landscape-scale CH_4_ emissions while also supporting ecosystem cobenefits such as wildlife habitat, biodiversity, flood mitigation, nutrient retention, and carbon sequestration (*18*, *19*).

The Global Methane Pledge, which was supported by more than 100 countries at COP26, aims to decrease anthropogenic emissions by 30% from 2020 levels by 2030 (*4*, *5*). The underlying assumption of the Pledge is that cuts in anthropogenic CH_4_ emissions will lower atmospheric CH_4_ concentrations relatively quickly due to methane’s short atmospheric lifetime (~10 years) (*3*). Our analysis of future CH_4_ emissions from PPR wetlands is a case study that demonstrates how natural CH_4_ emissions from wetlands will likely increase under future warming. Moreover, the increase in natural CH_4_ emissions is substantially greater under SSP5-8.5 warming compared to that under SSP2-4.5, highlighting the consequence of near-term policy decisions on long-term wetland CH_4_ emissions. Failing to account for increases in CH_4_ emissions from natural sources risks miscalibrating anthropogenic CH_4_ reduction targets. Improved estimates of both natural and anthropogenic sources of CH_4_ are needed to assess alternative climate mitigation strategies. Our analysis of wetland CH_4_ emissions provides us a framework for understanding how fine-resolution regional CH_4_ models can be used to improve coarser global models and provide scalable estimates to help decision-makers manage landscape CH_4_ emissions.

## MATERIALS AND METHODS

### Study area

The PPR (41.7° to 54.7°N latitude, 92.5° to 114.5°W longitude) covers ~820,000 km^2^ of the Great Plains in the United States and Canada and is home to millions of depressional wetlands (*16*). These depressions were formed during the Wisconsin glaciation, approximately 12,000 years ago (*17*). The underlying glacial till has low permeability, allowing depressions to fill and hold water and to ultimately develop into palustrine and lacustrine ecosystems made up of wetlands, ponds, and shallow lakes. We collectively refer to these depressional waterbodies as “wetlands” because the majority of them are less than 1 ha (0.01 km^2^) in size, less than 1 m deep, and seasonally (nonpermanent) ponded, meeting the definition of a wetland (sensu the Cowardin classification system) (*61*). In the PPR, larger wetlands often are shallow (<2 m) and have similar biogeochemical processes as smaller wetlands (*62*). Wetlands contain methanogenic microbial communities that are adapted to anoxic saturated soils, where they decompose organic material and produce CH_4_ (*9*). PPR wetlands range from fresh to hypersaline (up to three times more saline than the ocean) (*17*). This salinity is attributable to groundwater transport of dissolved sulfate through the ion-rich glacial till. PPR wetlands with higher sulfate concentrations have suppressed CH_4_ emissions (*23*), albeit high dissolved organic carbon concentrations in some PPR wetlands can support CH_4_ production in the presence of sulfate (*24*). Larger wetlands in topographically lower landscape positions tend to accumulate groundwater-derived solutes, while smaller, shallower wetlands are filled with rainwater and snowmelt (*45*). Vegetation characteristics of PPR wetlands are influenced by water depth and chemistry, typically with concentric zones with open water with submerged aquatic vegetation toward their centers and marshes and meadows with floating and emergent macrophytes such as *Typha* toward their edges (*37*, *44*).

The climate of the PPR is continental with a north to south temperature gradient ranging from ~2° to 8°C mean annual temperature and a northwest to southeast precipitation gradient ranging from ~400 to 900 mm year^−1^ (*63*, *64*). The PPR is centered on the confluence of tropical Pacific Ocean (El Niño–Southern Oscillation), eastern Pacific Ocean (Pacific Decadal Oscillation), and North Atlantic (Atlantic Multidecadal Oscillation) oscillations (*65*). Synergistic effects from synchronization of these oscillations lead to decadal periods of drought and deluge that influence groundwater levels. Wetland surface water is also extremely sensitive to variability in seasonal and annual precipitation (*66*). Thus, the size and hydroperiod of PPR wetlands are the result of complex interactions between long-term climate and short-term weather. An extreme multiyear drought from 1988 to 1992 led to the drying of many wetlands, with 1991 having the lowest wetland extent (*45*). Following the extreme drought, much of the PPR experienced a shift to a wetter climate. Annual precipitation in 2011 was one of the highest on record, and 2011 had some of the highest numbers of wetlands with ponded water (*45*). Therefore, we use 1991 and 2011 as extreme dry and wet years, respectively, in our modeling.

The PPR is intensively used for agricultural crop and biofuels production, which has led to extensive wetland drainage and upland conversion from prairie grassland to cropland (*16*). Drainage reduces CH_4_ emissions and soil organic carbon stocks but increases CO_2_ and nitrous oxide (N_2_O) emissions (*22*, *67*). Upland conversion from grassland to cropland can affect CH_4_ emissions indirectly through increased nutrient loading, resulting in increased CH_4_ emissions (*38*). However, wetlands nested in croplands are also subject to tillage and aeration of soils, thereby lowering CH_4_ emissions (*22*). Wetland drainage often results in consolidation of water from multiple smaller wetlands into one larger wetland. These larger wetlands are deeper with fewer fluctuations in water levels, as well as greater coverage of invasive emergent hybrid cattail, all of which favors CH_4_ production and emissions (*37*). Consolidation of wetlands also targets the smallest wetlands changing the distribution of wetland size classes, albeit the vast majority of wetlands are still <1 ha.

### Chamber (plot-scale) model: Data, sampling protocol, and flux calculations

The U.S. Geological Survey, Northern Prairie Wildlife Research Center has conducted and disseminated multiple greenhouse gas studies in the PPR (*21*, *22*, *62*, *68*). These studies used identical sampling designs and protocols using the static-chamber method to measure greenhouse gas flux and were combined in Tangen and Bansal (*69*). In the current analysis, we used only CH_4_ flux measurements associated with samples collected in the wetland zone of undrained wetlands during the growing season (*70*). This dataset of nearly 19,000 flux measurements was collected from 143 wetlands over 13 years of sampling, across an area of 200,000 km^2^. The expansive nature of our data covers a wide range of abiotic, biotic, and land-cover conditions that are representative of the PPR (*21*, *22*, *39*, *62*, *68*).

Methods for gas flux measurements are detailed in previous studies (*22*, *62*, *68*) and summarized here. Gas samples were collected along one transect per sampled wetland. Each transect had eight sampling locations: three in the upland zone and five in the wetland zone. Wetland zone chambers were spaced equally from the wetland edge to the wetland center (or up to a water depth of 2 m). At each sample location, chamber collars were inserted into the sediment if water levels were <5 cm depth; otherwise, chambers were deployed on floats.

Gas samples were collected every ~2 weeks, between 10:00 and 14:00, from May to November. Field measurements of greenhouse gas fluxes using the static chamber method are often conducted during the daytime hours when flux rates may be relatively high (*28*). Therefore, upscaling using chamber flux data may overestimate total emissions when fluxes are scaled to the 24-hour day. In the PPR, although diurnal variation in CH_4_ flux was observed in wetlands, the time when flux data were collected for this study was representative of average daily flux rates (*71*).

Opaque gas-collection chambers (20 cm in diameter and 20 cm in height) were placed onto the collars or floats. Ambient air samples were collected to approximate initial gas concentrations. After 30 min, headspace gas was collected, transferred to preevacuated vials, and transported to the laboratory. Concentrations of CH_4_, CO_2_, and N_2_O were measured on a gas chromatograph (SRI Model 8610, California, USA) interfaced with electron capture and flame ionization detectors. The detectors were calibrated with certified standard gas mixtures. CH_4_ flux rates were calculated using the linear change in CH_4_ concentration during the deployment, chamber dimensions (height and volume), and the ideal gas law (*25*, *68*). Pilot studies showed linear increases over a 30-min period. Because of potential nonlinear changes in gas concentrations, it is possible that our assumption of linearity may have underestimated flux (e.g., chamber gas concentrations equilibrate with the subsurface and asymptotes) or overestimated flux (e.g., if ebullition was artificially induced from sediment disturbance during deployment). To offset reduced accuracy of individual measurements, we used tens of thousands of measurements over space and time to improve our characterization of functional relationships between CH_4_ fluxes and explanatory variables (i.e., the “rule of large numbers”). Variables measured at each sample location during each sample event included water depth above the sediment surface, surface soil temperature (when there was <10-cm standing water) or water temperature (when water depth was >10 cm) using a 10-cm probe, air temperature, and surface soil volumetric water content (VWC, %) using a 5-cm probe. We assumed that water temperature is representative of soil surface temperature. WFPS was calculated by dividing VWC by soil porosity (*22*). Wetland size was determined by conducting detailed topographic surveys using Real-Time Kinematic Global Positioning System (RTK-GPS) of each wetland, creating digital elevation models, and determining wetland size based on the extent of wetland vegetation (see the Supplementary Materials).

### Chamber (plot-scale) model: Independent variables

In total, we analyzed 18,803 CH_4_ flux measurements and independent variables (*70*). The independent explanatory variables included WFPS (percent), soil temperature (degrees Celsius), wetland size (square meters), hydroperiod (days), surrounding land cover (grassland or cropland), remotely sensed NDVI values, and growing season interval (first “early” or second “late” half). We also included first-order time-lagged variables for WFPS, soil temperature, and NDVI. Detailed explanations for our choices and development of chamber model independent variables can be found in the Supplementary Materials.

### Chamber (plot-scale) model: Statistical details

We used a generalized additive modeling (GAM) approach to analyze the relationships between CH_4_ flux and our independent variables. We chose GAM because our data showed clear empirical nonlinear relationships between CH_4_ flux and our potential explanatory variables. GAMs have the capability of capturing nonlinear relationships and treating the degree of nonlinearity as a quantity to be estimated (*72*). Our inspection of the distribution of CH_4_ flux showed that CH_4_ flux was right skewed with some small negative values and a few extremely high values, which is common in wetland CH_4_ flux datasets (*50*). After consideration, we decided to focus only on positive flux values, which we assumed were log-normally distributed. Wetland-scale CH_4_ models have shown a negligible impact on CH_4_ emissions when negative fluxes are excluded as long as inundation variability is considered (*73*).

We fit a GAM to our data using a penalized spline regression algorithm implemented in the mgcv package in the R (version 4.0.5) programming environment (*74*). This algorithm estimates spline terms on the continuous parameters as part of the model fitting process by balancing model fit against overall smoothness. The degree of smoothness is often reported as the effective degrees of freedom, which can often be interpreted as the number of polynomial terms needed to obtain a similar estimate with a generalized linear model. We treated “surrounding land cover” and “growing season interval” as categorical variables and assumed spline terms on all the continuous main effects in the model. Because observations within a year came from repeated measurements at each wetland and each chamber within each wetland, we assumed a repeated-measures model structure when fitting the model. This entailed adding “wetland” and “chamber” within each wetland as random effect terms in the model. The mgcv package allows for the inclusion of random effects as additional spline terms in the model.

The mgcv package also includes a built-in variable selection capability that uses a penalty term on the likelihood function. The fitting algorithm is allowed to set this penalty term to zero for main effects if they do not contribute to a maximized likelihood, which effectively removes those variables from the model. We used this selection process in our model fitting and allowed the algorithm to remove any of the smoothed main effect variables from the model. Categorical variables were not removed in the fitting process. We fit the full model with all fixed effects, and the selection function included all variables in the final fit. This model had an adjusted *r*^2^ = 0.62 and deviance explained of 63%. The functional relationships between model variables and CH_4_ flux are shown in Fig. 2.

To inform biogeochemical models that use *Q*_10_ coefficients of CH_4_, CO_2_, and the CO_2_:CH_4_ ratio, we computed *Q*_10_ for both gases using the *Q*_10_ function in the respirometry package in R (*75*) using mean values of field-measured gas fluxes for each 1°C increment of air or soil temperature (measured in the top 10 cm of soils). *Q*_10_ varies by temperature range and is sensitive to hydrologic conditions; therefore, we calculated *Q*_10_ over all air and soil temperatures and for each 10°C increment (all, 0° to 10°C, 10° to 20°C, and 20° to 30°C) using data across all hydrologic conditions as well as a subset of saturated conditions in which WFPS was 100% (table S1).

### Landscape model: Predictors

To spatially and temporally upscale modeled CH_4_ flux patterns to the entire PPR over multiple climate conditions and scenarios, we first required a series of spatially explicit, temporally dynamic rasters of relevant predictors that were surrogates of the explanatory variables used in our GAM. The GAM revealed how fine-scale spatial and temporal variation in predictors can have nonlinear impacts on CH_4_ fluxes. We sought to use these fine-scale predictors to model historical conditions that represented extremely dry and wet periods (1991 and 2011, respectively). The finest spatial resolution that we could model, given available remotely sensed data for the PPR dating back to 1991, was 30 m based on Landsat satellite imagery.

For each variable in the GAM model, we acquired or modeled remotely sensed predictors that represented each chamber variable at the scale of the PPR. We used the DSWE algorithm to determine the presence, permanence, and extent of water to represent WFPS, hydroperiod, and wetland size, respectively (*47*). The downscaled Parameter-elevation Relationships on Independent Slopes Model database was used for temperature, and the North American Land Change Monitoring System was used for surrounding land cover. Additional detailed descriptions of landscape model predictors can be found in the Supplementary Materials.

Remotely sensed predictors were aligned with the set of years 2003 to 2016 and the locations corresponding to our field measurements. We then fit a landscape model using an RF algorithm (described below). Remotely sensed predictors were also generated for the years 1991 and 2011 over the entire PPR. We then used these predictors as inputs to the landscape model to estimate historical PPR wetland CH_4_ fluxes. We also used spatially explicit monthly temperature projections from two climate scenarios (SSP2-4.5 and SSP5-8.5) as model inputs to estimate future CH_4_ fluxes. For each climate scenario, temperature values were based on an ensemble of 13 ESMs from the Coupled Model Intercomparison Project Phase 6 (CMIP6) designed for robust downscaling of monthly climate normals (2081 to 2100). These 13 selected ESMs are representative of the distribution of equilibrium climate sensitivity, which include ACCESS-ESM1-5, BCC-CSM2-MR, CNRM-ESM2-1, CanESM5, EC-Earth3, GFDL-ESM4, GISS-E2-1-G, INM-CM5-0, IPSL-CM6A-LR, MIROC6, MPI-ESM1-2-HR, MRI-ESM2-0, and UKESM1-0-LL (*54*, *76*). A preliminary analysis showed that monthly temperature projections from the mean of the 13-model ensemble had the least difference from observed historical temperature for the central PPR. Each of the 13 models was run individually to quantify the mean and range of CH_4_ predictions among models.

### Landscape model: Statistical details

We used RF regression as our method of upscaling estimated CH_4_ fluxes to the landscape (*77*–*79*). RF models aggregate the results of ensemble regression trees (creating a “forest” of individual decision trees) that are each generated using a randomly bootstrapped training dataset (~^2^/_3_ of data) and a random subset of predictors at each split node (*80*). Then, the model is evaluated using the data that were not used (~^1^/_3_ data) in parameterizing the model. The high accuracy of RF is based on the assumption that many uncorrelated models will outperform individual models (*80*). Like GAMs, RF models can detect nonlinear relationships between variables while guarding against overfitting (*78*, *80*). RF models also tend to have higher predictive accuracy than other ML algorithms when modeling CO_2_ and CH_4_ fluxes (*77*, *78*, *81*).

Unlike many other upscaling efforts that use ML for evaluating dozens to hundreds of predictor variables, our approach involved a priori selection of predictors based on our analysis of the explanatory variables identified as important drivers of CH_4_ flux in the GAM chamber model. Using this approach, we were able to compare the estimated relationships between the GAM and RF models to (i) ensure that the functional relationships between explanatory and response variables were similar between the two modeling approaches, which then allowed us to (ii) run more realistic scenarios based on how potential changes in environmental predictors could affect CH_4_ fluxes. We selected input data for the RF model that included a mix of field-measured data and remotely sensed predictors, which has been shown to improve models more than either data source alone (*79*). More details on development of the RF model can be found in the Supplementary Materials. The RF model explained 61.3% of the variance and had a mean squared residual of 4.65 and a negligible bias (fig. S4).

### Landscape model: Predictions

We used our spatially explicit predictors and RF model to make predictions of CH_4_ flux for all wetland pixels in the entire PPR for two historical dry and wet periods (1991 and 2011, respectively) to understand the range of natural variation in CH_4_ fluxes. We focused on predicting growing season fluxes. Studies of wetland CH_4_ flux during winter months have demonstrated the importance of nongrowing season emissions to annual CH_4_ budgets (*48*–*50*). Ice breakup and its thaw in spring potentially may be hot moments for release of physically trapped and accumulated CH_4_ under ice over the winter (*51*). However, our wetlands had low nongrowing season flux rates (median, 0.007 mg of CH_4_ m^−2^ hour^−1^) (*69*), as did an inversion study in the same region (*82*). Therefore, we assumed that the predicted CH_4_ flux rates from the first and last time steps of the frost-free season represented winter rates before and following the frost-free season, respectively. Exclusion of nongrowing season dynamics, especially the physical processes of freeze up and thaw in the shoulder seasons, may have underestimated our cumulative CH_4_ flux estimates.

We also developed models for four future climate scenarios to examine how different assumptions about warming and wetland extent may affect CH_4_ fluxes by 2100. Our current analysis only focuses on climate change scenarios and not land use scenarios. In a study that compared 11 future land use scenarios in the Northern Great Plains, there was a wide variability in future cropland extent with both gains and losses and an increase in grassland extent for cellulosic biofuels production (*40*).

Wetland extent has been identified as a primary source of uncertainty in CH_4_ fluxes (*2*, *32*). Unlike the clear consensus among ESMs in the directionality of future atmospheric temperature warming, there is much less agreement among forecasts of future precipitation in the PPR. Future mean annual precipitation change in the PPR by the end of the 21st century could range from −19 to +33% when compared to 1990 to 2020 normals (*55*). Therefore, to simulate wetland extent of future scenarios, we used DSWE and NDVI rasters from 1991 and 2011 to represent bookends of the potential range in wetland extent from dry to wet, respectively, in the PPR. For future temperatures, we used a 13-model ensemble of ESMs (described previously) associated with SSP2-4.5 (moderate warming, ~1.7°C) and SSP5-8.5 (severe warming, ~2.7°C) for the period 2081 to 2100. We combined our dry and wet bookends with the two warming regimes to create four future scenarios: (i) moderate warming and dry, (ii) moderate warming and wet, (iii) severe warming and dry, and (iv) severe warming and wet.

In total, our upscaling efforts covered ~3.8 trillion pixels [2 historical climate conditions × (4 future climate scenarios × 13 ESMs) × 26 time steps per condition or scenario × 1.4 billion pixels in the PPR]. Because predicting CH_4_ flux under these scenarios was extremely computationally expensive, we limited our analyses to these two historical conditions and four future scenarios. Additional details on the landscape model are provided in the Supplementary Materials.

### Landscape model: Global model comparisons

To compare our CH_4_ predictions to those from global models, we acquired monthly predictions from the 13 bottom-up, biogeochemical process-based models and 21 top-down inversion models used in the GCPv2 and from the 18-member WetCHARTs v1.3.1 models (*52*, *83*). For the 13 bottom-up GCPv2 models, we made additional comparisons between estimates of CH_4_ using the WAD2M wetland maps versus model-specific maps (when available) for wetland extent (table S2) (*53*). The global estimates for each of the 52 models (13 GCPv2 bottom-up, 21 GCPv2 top-down, and 18 WetCHARTs) were subset to the year 2011, cropped and masked to the PPR (fig. S3), scaled to Tg of CH_4_ (table S2), and compared to our 2011 PPR estimate of 0.62 Tg of CH_4_. We recognize that there are more complex methods for spatial and temporal model comparisons; however, those analyses are beyond the scope of this study.

To compare wetland extent between our PPR model and WAD2M, we used the WAD2M monthly inundation data from 2000 to 2017 at the 0.25–arc-degree resolution (*53*). We subset inundation data for June, July, and August of the year 2011, extracted the pixels with maximum wetland extent among the 3 months, scaled the fraction of inundated per pixel to square kilometers, and then summed these values over the PPR. This method provided a wetland extent in the PPR that was most similar to our protocol for determining wetland extent in the PPR using DSWE.

## Supplementary Materials

### This PDF file includes:

Supplementary Text Figs. S1 to S4 Tables S1 to S3 References
