# Supplementary material for: Large increases in methane emissions expected from North America’s largest wetland complex
Source: Sci Adv. 2023 Mar 1;9(9):eade1112. doi: 10.1126/sciadv.ade1112 (PMC9977182; doi:10.1126/sciadv.ade1112)
Supplement: Supplementary file 1 — Supplementary Text Figs. S1 to S4 Tables S1 to S3 References [file sciadv.ade1112_sm.pdf]

Science Advances
AAAS

Supplementary Materials for

Large increases in methane emissions expected from North America's largest wetland complex

Sheel Bansal *et al.*

Corresponding author: Sheel Bansal, sbansal@usgs.gov

Sci. Adv. **9**, eade1112 (2023)
DOI: 10.1126/sciadv.ade1112

This PDF file includes:

Supplementary Text
Figs. S1 to S4
Tables S1 to S3
References

Supplementary Text

Chamber model: Developing independent variables

We developed a chamber (plot-scale) model of the relationships between wetland methane (CH_4) flux (in mg m^{-2} hr^{-1}) and explanatory independent variables in the Prairie Pothole Region (PPR). The variables we selected were either supported by theory or from empirical relationships in our data.

We first considered water-filled pore space (WFPS) to be among the more important explanatory variables in our analysis. Methanogenesis typically occurs under anoxic conditions, which occurs when soil saturation is sufficiently high to limit the supply of oxygen. Methanogenesis can also occur in anaerobic microsites in drier soils or in oxic water columns (*86*). We observed that the saturation point at which CH_4 emissions spiked occurred around 80% WFPS (Fig. 2A). When soils transition from ponded to unponded, there is a lag period in which methanogenesis and CH_4 emissions continues because CH_4-producing anoxic zone may persist below the sediment surface (*87*). Also, alternative electron acceptors used in anaerobic respiration such as Fe (III) and humic acids take time to re-oxidize during drying. Conversely, when soils transition from unponded to ponded, there is a lag period until oxidation-reduction reaction (redox) conditions are sufficiently low to favor methanogens. Not accounting for these lags could result in underestimates or overestimates of CH_4 flux (*28*). Thus, we also considered WFPS measured during the previous flux measurement, approximately two-weeks earlier (WFPS-lag), as an explanatory variable.

Longer periods of time above the saturation point, usually during ponded conditions, promote greater CH_4 emissions. Therefore, we considered the number of days that soils were ponded over the frost-free season as an explanatory variable in the model, which we referred to as 'hydroperiod'. Hydroperiod was calculated for each chamber in each year as the number of days a chamber was in a ponded (water depth > 0 cm) condition. We assumed chambers remained ponded for all days (~14) between consecutive sampling events that both had water depths > 0 cm. Similarly, we assumed chambers were unponded for all days between consecutive sampling events where both water depths were 0 cm. We assumed that chambers were ponded for half the days between consecutive sampling events when the chamber transitioned between water depth > 0 and 0 cm.

Warm temperatures can stimulate CH_4 production (*9, 10*). Methanogenesis is a microbial process that follows a classic Arrhenius metabolic response (i.e., cellular activity increases exponentially with temperature under saturated conditions; Table S1) with an optimum around 25 to 30 °C (*88, 89*). Therefore, we also included soil temperature as a potential explanatory variable. Because CH_4 produced in sediment can take time to diffuse to the surface, we also considered soil temperature from the previous measurement (soil temperature-lag) in the model.

Increased carbon substrate availability later in the growing season, when plants are fully grown, allows methanogens to produce more CH_4 per unit temperature (*90*). Microbial response to temperature can also change over the growing season (*91*). Using eddy covariance CH_4 flux data from several wetland types, Chang et al. (*90*) demonstrated how CH_4 emissions were often higher per unit temperature later in the growing season, presumably when methanogens switch from hydrogenotrophic to acetolactic production pathways (*92*), leading to higher CH_4 emissions in the second compared to first half of the growing season. To account for seasonality in our analysis, we considered the 'growing season interval' in the model. We categorically defined this interval according to days prior to ("early") or following ("late") the day of maximum temperature. To determine the day of maximum temperature, we fitted a quadratic function between field-measured soil temperature and day of year (DOY). The day of maximum soil

temperature was the DOY that corresponded with the maximum fitted temperature value from the quadratic function.

Methanogenesis is highly sensitive to primary productivity because plants provide carbon substrates to fuel microbial activity and act as transport pathways through their stems (*9, 35, 93*); thus, vegetation biomass and phenology have been linked to CH_4 emissions and seasonal patterns (*94*). Wetlands in the PPR can vary considerably in terms of primary productivity, with some wetlands dominated by dense emergent macrophytes and others having floating, submerged, or no vegetation. Wetlands with more vegetation and photosynthetic activity are assumed to produce more CH_4 because of higher carbon inputs into the soil (*28*), and therefore vegetation is typically included in wetland CH_4 models (*95*). One measure of primary productivity is the Normalized Difference Vegetation Index (NDVI), which is computed from spectral imagery. NDVI is one of the most widely used vegetation indices (*96–98*). Like most indices, the NDVI transforms reflectance measurements based on the reflectance peak of vegetation in the near-infrared (NIR) and red wavelength ranges where chlorophyll absorbs light energy for photosynthesis. NDVI is often used as a reliable measure of 'greenness' or leaf area index and values theoretically range from -1 (no greenness, generally in surface water void of plant or algal life) to 1 (high greenness, in dense vegetative canopy) (*99*). We acquired NDVI from Landsat imagery (described below) that corresponded with each CH_4 flux sampling event and location to include as a potential explanatory variable in our chamber model. Similar to WFPS and soil temperature, we considered 'NDVI-lag' in the model to account for a lag time for plant-derived carbon substrates to become available for methanogenesis.

Soil organic carbon (SOC, %) was also considered for the chamber model as another indicator of carbon substrates for methanogenesis. However, our one-time measurement of SOC was not a strong independent predictor of CH_4 flux. Wetlands nested in grasslands with minimal disturbance had relatively high SOC compared to wetlands in agricultural fields. Therefore, we used 'surrounding land cover' as an independent variable that also served as a surrogate for SOC (see description of land cover below).

There are a number of studies showing that CH_4 fluxes increase with decreasing waterbody sizes (*26, 41, 42, 100*), which is attributable to a number of mechanisms. For example, smaller, shallower wetlands can have 'wall-to-wall' coverage of dense macrophytes (which promotes CH_4 production and transport), whereas vegetation in larger wetlands and lakes is often restricted to wetland edges. Larger wetlands also have lower perimeter to area ratios, which limits the amount of wetland surface area that is in direct contact with CH_4-rich sediment (*42*). These larger wetlands are also likely to receive relatively more groundwater inputs (*101*), which results in higher levels of salinity that can inhibit methanogenesis (*17, 62, 66, 102*). Additionally, smaller wetlands tend to have higher concentrations of dissolved organic carbon to fuel methanogenesis (*24, 103*). Thus, we included wetland 'size' as a potential explanatory variable.

Wetland size was determined by conducting detailed topographic surveys of each wetland using an RTK-GPS surveying system (Trimble 5700, Trimble, Sunnyvale, California, USA). Surveys consisted of high-density logging of points throughout the wetland catchment, as well as delineating and logging features such as vegetation zone boundaries. The survey data were imported into the software program ForeSight (Tripod Data Systems, Corvallis, Oregon), where a digital elevation model was used to determine wetland size based on the extent of wetland vegetation (i.e., elevation of vegetation boundary survey points).

The explanatory variables we have considered, thus far, describe both general and wetland-specific attributes that are correlated with CH_4 flux. Many of the wetlands in the PPR are subjected to management when nested in croplands, including tillage and planting of crops when soils are sufficiently dry. These management practices decrease wetland CH_4 fluxes through soil aeration and loss of soil organic carbon (*39, 58*). At the same time, nutrient loading into wetlands from agricultural runoff can lead to increases in CH_4 emissions (*38*). PPR wetlands are also

drained and consolidated into fewer, larger wetlands. Consolidated wetland often have more emergent vegetation (e.g., more *Typha* × *glauca*) and geometries that are more compact with lower perimeter to area ratios (*104, 105*). In the PPR, each of the major crops in the USA (e.g., corn, soybean, wheat, barley) and Canada (e.g., canola) have different management practices and therefore may have differing effects on CH_4 fluxes. Our field data has incomplete descriptions of management activities and there are limited spatially explicit map on crops and field management that is consistent between the USA and Canada. Therefore, we simplified the 'surrounding land cover' variable as either: 'cropland' or 'grassland'. Flux measurements from native prairie (n = 9,100), hydrologically restored (n = 4,373), and grassland restored (n = 2,794) prairie sites were binned and categorized as 'grassland'; fluxes from cropped wetlands with intact hydrology (i.e., not drained, n = 3,325) were categorized as 'cropland'. Drained wetlands were excluded from the analysis. Aside from lagged variables, there was limited correlation among independent variables ($r^2 = 0.53$ between WFPS and hydroperiod; $r^2 = 0.43$ between soil temperature and NDVI; all other r^2 between independent variables were below 0.30).

Landscape model: Developing remotely sensed predictor variables

In order to make landscape-level predictions across the PPR with our model, we needed to develop remotely sensed surrogates for our field measurements of WFPS, hydroperiod, and wetland size. We used the Dynamic Surface Water Extent (DSWE) algorithm to classify pixels on the landscape in terms of presence, permanence, and extent of water. The DSWE classifier was developed by the U.S. Geological Survey and is used to generate an operational surface water product that is publicly available through the Earth Resources Observation and Science (EROS) Center. This classifier uses a series of five tests to identify surface water for each clear (cloud-, shadow-, and snow-free) pixel in the Landsat archive (*47*). The result is a quality-assured, terrain-corrected classification of each pixel into one of five categories: 0 (Not water), 1 (Open water – high confidence), 2 (Open water – moderate confidence), 3 (Partial surface water/wetland – conservative), and 4 (Partial surface water/wetland – aggressive). DSWE is ideal for the treeless, relatively flat terrain of the PPR because it identifies both open waters (typically DSWE class 1 and 2) and vegetated wetlands (typically DSWE class 3 and 4), which describes the majority of PPR wetlands. In contrast, other globally gridded products of surface water, such as from the European Commission's Joint Research Centre Global Surface Water Explorer dataset (*106*), primarily capture open water pixels. Even so, despite 30-m resolution offering a unique opportunity to capture fine-scale CH_4 emissions, many wetlands in the PPR are smaller (e.g., 10-m) and were not remotely sensed. However, our model results show that CH_4 emissions dramatically decrease in the smallest wetlands (< 0.1 ha), and therefore we assume that exclusion of these wetlands did not have a large influence on total annual CH_4 emissions estimates. Future modeling efforts for CH_4 fluxes that only focus on recent years (i.e., 2015 onward) could improve estimates using newer satellite information (e.g., synthetic-aperture radar from Sentinel-1).

All landscape rasters were cropped and masked using a PPR shapefile from the USGS (*107*) and were in the Albers Equal Area projection. We removed major rivers and large, deep lakes from our model due to different mechanisms driving CH_4 fluxes and lack of data to parameterize those systems (USA and Canada Water Polygons (*108*)). For the purposes of this study, we considered any pixel classified in DSWE class 1 to 4 as surface water capable of producing CH_4. We calculated the average WFPS associated with each DSWE class using field measured data (0 = 91.45%, 1 = 99.84%, 2 = 98.41%, 3 = 98.29%, 4 = 93.71%; used in Fig. 3C).

Landsat data served as our remote sensing input for developing DSWE rasters. Image capture frequency for Landsat is either 8 or 16 days. Because we limited Landsat observations to Scanline-Corrector-On (SLC-on) scenes only, we used 8-day images up through May 2003 and 16-day images after this date (that is, during the period the Landsat 7 ETM+ Scanline Corrector

was off). The result was a partial hydrological history for each pixel in the study area due to the presence of clouds, cloud shadow, or snow that prevented proper hydrologic classification of all pixels for all two-week timesteps. To address data gaps, we used harmonic regression to model trends in input indices for each pixel and synthesize a full hydrological history. Harmonic regression, also referred to as trigonometric or cosinor regression, is a linear regression model in which the predictor variables are the trigonometric functions of the predicted variable (*109, 110*). This model is typically used in situations where the pattern of the predicted variable follows a temporal cycle, such as those caused by seasonal biological processes. Harmonic regression has been used to re-construct time series data in imagery captured by a wide array of sensors (e.g., Landsat TM, ETM+, OLI, MODIS, NOAA-7) (*111–115*). Previous studies have used raw and derived imagery components (e.g., brightness, NDVI, NIR reflectance) as independent variables in harmonic regression models to project landcover, forest types, canopy cover, biomass, and crop pest damage (*116–120*). We used time (as Julian date) as a predictor variable in the harmonic regression.

For each year of flux measurements (2003–2016), we fit the harmonic regression model using Google Earth Engine (GEE) and the GEE Landsat library (4-5, TM, ETM+ [SLC on only], and OLI), to produce DSWE classifications using code from Soulard et al. (*85*). We exported the resulting DSWE-classified raster for each two-week timestep of the year (26 rasters for each year). We created a time series raster by stacking and compositing the 26 raster layers in chronological order. The result was a time series composite image comprised of 26 bands, each band corresponding to a two-week timestep. We then fed the 26-band composite images into R (*121*) as raster stacks (i.e., time series) and extracted 'DSWE' and 'DSWE-lag' values that corresponded with the dates and locations of each flux measurement (*70*). We also generated PPR-wide DSWE raster stacks for 1991 and 2011, which we used for estimating landscape-scale CH_4 fluxes. DSWE values during periods of snow or ice are meaningless, and therefore we converted all DSWE values to class zero for days where the air temperature was < 0 °C. We generated and processed 'NDVI' and 'NDVI-lag' raster stacks using the same method as for DSWE (*70*).

Hydroperiod was calculated per pixel by summing the number of two-week timesteps during the frost-free season (air temperature > 0 °C) that a pixel had water (any DSWE class 1–4) and multiplying that value by 14 days. Wetland size was determined by first generating a single wetland mask layer of all pixels that had water (any DSWE class 1–4) during timesteps from the beginning of June through the end of August (layers 12 through 17). Connected water pixels were combined into distinct clumps (i.e., unique wetlands) using the *clump* function from the *raster* package (*122*) in R (similar to polygonizing). We then computed wetland size (in m^2) of each clump by summing the number of pixels in each clump and multiplying by the resolution of each pixel. We tallied all unique clumps to estimate total number of wetlands (Table S3). Unique year-specific wetland masks were generated for each year of sampling. Similarly, year-specific wetland masks for the PPR were generated for each of the two historical conditions (1991, 2011) (*70*).

To develop the predictors 'soil temperature' and 'soil temperature-lag', we used spatially explicit air temperature data from the Parameter-elevation Relationships on Independent Slopes Model (PRISM) database. PRISM provides monthly air temperature data scaled to 30-arc second grids (800 m) using locally weighted weather station data (*123*). The open-source program ClimateNA version 7.20 was used to downscale PRISM data to Landsat 30-m pixel resolution (*64*). To rescale the monthly air temperature values from ClimateNA to match our two-week prediction timesteps, we fit a spline through the monthly ClimateNA values to predict temperature values for every 14th day throughout the year, which was used as the temperature value of each two-week timestep. Temperature values in each timestep were merged with our field-measured flux database based on date and location. Because PRISM supplies air temperature

values, we needed to adjust these values to be more consistent with the soil temperature values we measured in the field. To accomplish this, we modeled soil temperature using generalized additive modeling (GAM) to develop a function that related field-based soil temperature measurements to downscaled PRISM air temperature data ($r^2 = 0.70$). The range of temperature values used to build the random forest model spanned the complete range of temperature values used for spatial predictions under Socioeconomic Pathways (SSP) 2-4.5 and > 99% of the values under SSP5-8.5 climate scenarios, therefore minimizing the need to extrapolate beyond the temperature range of our input data. 'Growing season interval' was assigned to each two-week timestep as 'early' or 'late' based on the timestep with the maximum temperature of the year, similar to methods used in Change et al. (*90*). The two-week timestep that contained the maximum temperature for the year was classified as 'late'.

ClimateNA also provides future estimates of monthly temperature data using various Coupled Model Intercomparison Project Phase 6 (CMIP6) Earth System Models (ESMs) that we used in this analysis. Similar to PRISM air temperature, future air temperatures were also corrected to soil temperature using the GAM model. To forecast future CH_4 emissions, we used future monthly temperature projections from two climate scenarios (SSP2-4.5 and SSP5-8.5, see Materials and Methods for additional details).

For the 'surrounding land cover' predictor, we used a land use land cover (LULC) product~~,~~ published by the North American Land Change Monitoring System (NALCMS), a joint initiative between Natural Resources Canada/Canada Centre for Remote Sensing (NRCan/CCRS), the U.S. Geological Survey, and three Mexican organizations: The National Institute of Statistics and Geography (Instituto Nacional de Estadística y Geografía – INEGI), the National Commission for the Knowledge and Use of Biodiversity (Comisión Nacional para el Conocimiento y Uso de la Biodiversidad – Canabio), and the National Forestry Commission (Comisión Nacional Forestal – Conafor), and supported by the Commission for Environmental Cooperation (CEC). These data represent LULC at a 30-m resolution for the entirety of North America and therefore have the advantage of continuity across international boundaries as opposed to other land cover products representing only the USA or Canada (e.g., the National Land Cover Database in the USA). Each map product was generated using Landsat satellite imagery and consists of data classified into nineteen Level II land cover classes according to the Land Cover Classification System standard developed by the Food and Agriculture Organization of the United Nations.

We acquired the 2010 NALCMS raster product from the CEC data hub (*124*) and clipped it to the PPR in GEE. Of the nineteen land cover classes, four represent water or wetland LULC. To attribute a 'surrounding land cover' category to each wetland pixel as 'cropland' or 'grassland', we masked pixels classified in the four water or wetland classes and filled the masked data with the mode of a moving 6- × 6-pixel window (similar to nearest-neighbor). For instance, a pixel in the NALCMS raster classified as water or wetland would have its classified value changed to the most common LULC value of the six pixels surrounding it. The final raster was read into R and included as representative land cover for all climate scenarios (*115*). Using a consistent land cover raster among scenarios allowed us to isolate the effects of climate on CH_4 emissions.

The dataset used to build the landscape model was a subset of the published chamber flux dataset (*70*) that only included flux measurements that were captured in the wetland masks (n = 6,592). Similarly, we only predicted CH_4 flux for pixels that were included in the year-specific wetland masks.

Landscape model: Random forest model details

Both field measured data and remotely sensed predictors were used in the random forest (RF) model to predict landscape CH_4 emission. For field data, we used soil temperature, wetland size, and surrounding land cover due to higher accuracy of field measurements compared to remotely sensed data. For remotely sensed data, we used hydroperiod and growing season interval as those data require full growing season information, which was not always collected in the field particularly in years 2005, 2006, and 2008. We also used remotely sensed NDVI because no measure of vegetation was taken during field measurements. CH_4 fluxes were restricted to values greater than zero to focus on periods with sufficient CH_4 flux data. CH_4 fluxes were log transformed to improve fit for the majority of the data.

We trained our RF model using the *randomForest* package in R (*125*) with 150 randomized decision trees. The choice to use 150 trees was based on preliminary analyses indicating a saturation effect in the prediction performance over 150 trees. We also conducted a preliminary analysis to determine an appropriate number of random variables selected at each split to build the individual trees of the forest (referred to as 'mtry' in the *randomForest* package). Since we had 10 variables (N) in our model, we tested up to 10 mtry values, though N/3 is typically the optimal number for RF regressions. Higher mtry values increase accuracy while lower mtry values increase generality. We chose a value of 3 to favor relative generality of the model with minimal loss of accuracy from not using higher mtry values (e.g., mtry of 3 versus 4 explained 61% versus 62% of variation, respectively). We left the remaining parameters at their default values of the *randomForest* package as there were minimal changes in model performance when varying those parameters (e.g., minimum sample size of 5 in terminal leaves). Variable importance was determined using the permutation method. The dataset used to build the RF model had no missing values, so no imputation was required. Performance of the model was assessed by the mean of squared residuals, percent variance explained, as well as partial-dependency plots indicating the shape of the relationship between CH_4 flux rates and predictor variables (Figs. S1, S4).

We used the results from our chamber model to select which predictor variables to include in the RF model. We then used the *Boruta* package in R (*126*) to determine which of the predictors in the RF model were most relevant. This package works by comparing model performance using the original dataset against performance of a model built with a randomly shuffled dataset. The *Boruta* algorithm determined that all the predictors in the model were important.

<u>Landscape model: Summarizing annual methane emissions</u>

Annual CH_4 emissions were summed across all wetland pixels in the PPR to determine landscape wetland total annual CH_4 emissions for each historical condition or future climate scenario (Tables 1 and S3). To calculate annual cumulative flux per pixel, hourly flux rates were scaled to the day and then scaled to the two-week timestep and summed over the 26 timesteps. Predictor information, including mean soil temperature, mean NDVI, mean hydroperiod, total wetland extent, wetland count, and growing season length were also summarized for each climate scenario across the PPR and by location. Annual CH_4 values were also differentiated by size class (1.0 ha intervals from 1–10 ha and > 10 ha) and by country and their respective states (in USA) or provinces (in Canada) (Fig. 5). The percent of CH_4 emissions in each size class was calculated by dividing CH_4 emissions of each size class by total CH_4 emissions; similarly, the percent of wetland extent in each size class was calculated by dividing wetland extent of each size class by the sum total of all wetland extent (Fig. 5).

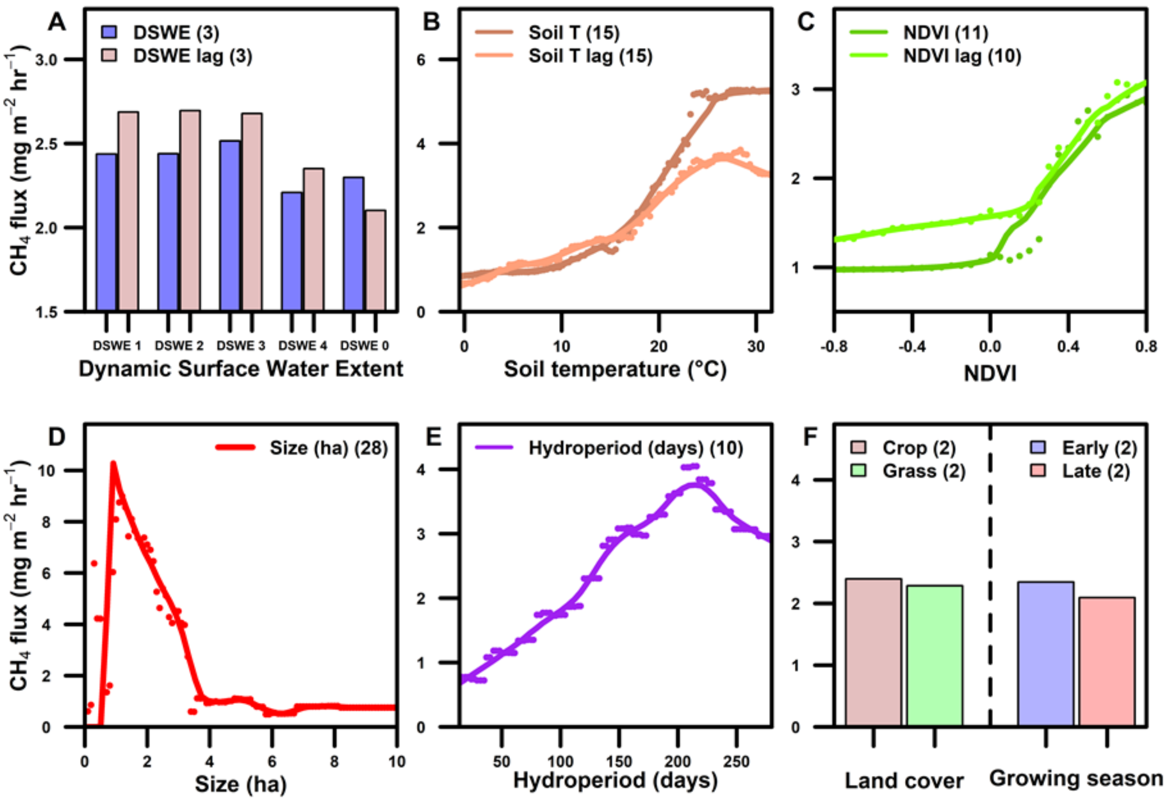

Fig. S1. Random forest model partial dependency plots of methane (CH_4) flux (mg m^{-2} hr^{-1}) and predictors for the Prairie Pothole Region of North America. Relations between CH_4 flux and (**A**) Dynamic Surface Water Extent (DSWE) and its lag (DSWE lag)*, (**B**) soil temperature (Soil T) and its lag (Soil T lag)* (degrees Celsius), (**C**) Normalized Difference Vegetation Index (NDVI) and its lag (NDVI lag)*, (**D**) wetland size (ha), (**E**), hydroperiod (days), (**F, left**) surrounding land cover, and (**F, right**) growing season interval (i.e., first [early] or second [late] half of the growing season). *Lagged predictors represent values from the prior 2-week timestep. Points in (**A–E**) represent modeled flux rates, lines represent loess smoothed relationships between a predictor and CH_4 flux. Numbers in parentheses represent the relative percent of variable importance.

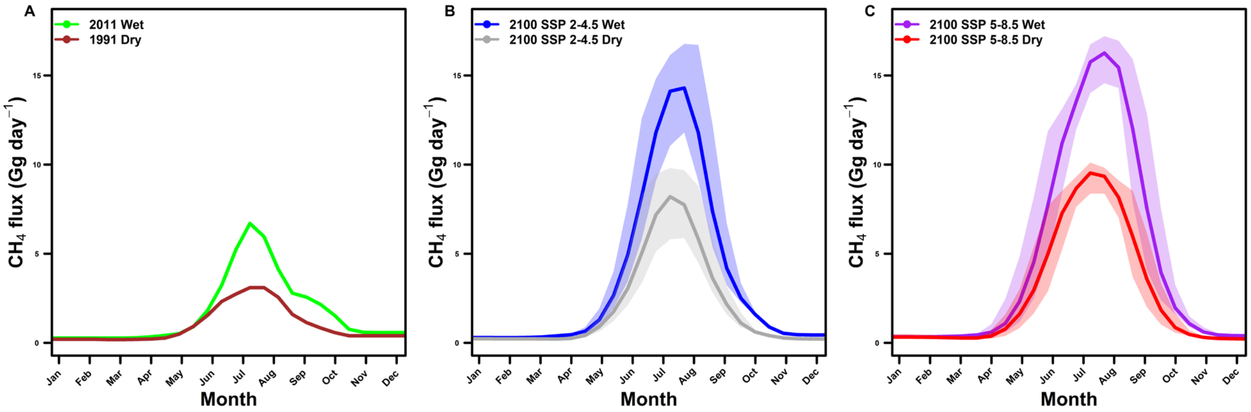

Fig. S2. Seasonal pattern of mean methane (CH_4) flux (Gg day^{-1}) under (A) two historical climate conditions and (B, C) future climate scenarios from the Prairie Pothole Region. (A) Lines represent mean flux rates under two historical climate conditions that represent extreme dry (1991, brown) and wet years (2011, green). (**B**) Two future (2100) climate scenarios under dry (gray) or wet (blue) potential hydrologic conditions with moderate (~1.7 °C) warming (Socioeconomic Pathways [SSP] SSP2-4.5). (**C**) Two future climate scenarios under dry (red) or wet (purple) potential hydrologic conditions with severe (~2.7 °C) warming (SSP5-8.5). Solid lines indicate means and shaded regions of future scenarios indicate range of predictions from 13 Earth System Models from the Coupled Model Intercomparison Project Phase 6.

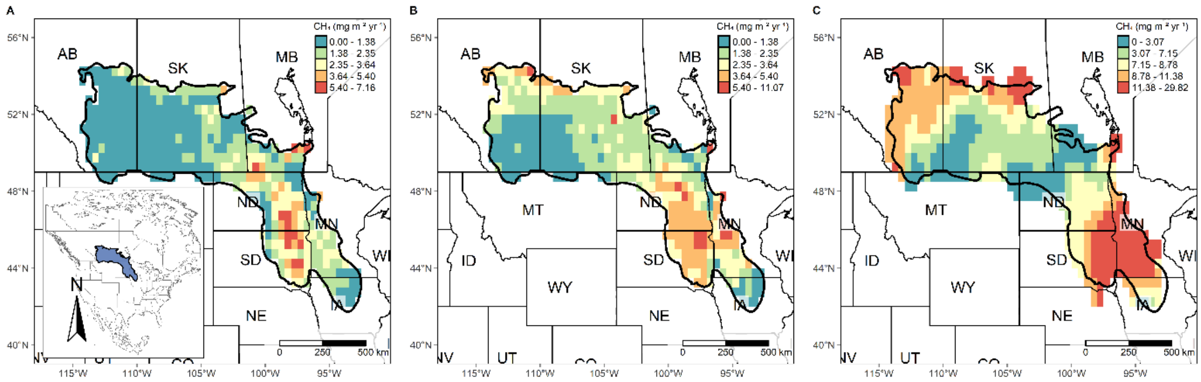

Fig. S3. Global model ensemble means of wetland methane (CH_4) emissions (mg m^{-2} yr^{-1}) in the year 2011 in the Prairie Pothole Region (PPR). (**A**) Ensemble of 13 bottom-up biogeochemical models of the Global Carbon Project version 2 (GCPv2). (**B**) Ensemble of 18-member bottom-up, process-informed models of WetCHARTs v1.3.1. (**C**) Ensemble of 21 top-down inversion models of GCPv2. Ensemble means of bottom-up GCPv2 models were calculated using WAD2M wetland maps. CH_4 flux rates are per meter square of the entire pixel (not per wetland area within each pixel). Bottom-up GCPv2 (**A**) and WETCHARTs (**B**) model results are both displayed in their native 0.5° resolution. The top-down model (**C**) result was resampled from its native 1.0° to 0.5° resolution to facilitate comparisons among maps. See Table S2 for total PPR annual CH_4 emission among models. Legend colors (quantiles) in **A** and **B** are the same, but are unique for map **C** to help visualize similarities in spatial distribution of CH_4 fluxes among models. Base map used in (**A–C**) from rnaturalearth: World Map Data from Natural Earth (https://docs.ropensci.org/rnaturalearth). Data to generate GCPv2 and WetCHARTs ensemble maps obtained from Saunois et al. (*52*) and Bloom et al. (*83*), respectively.

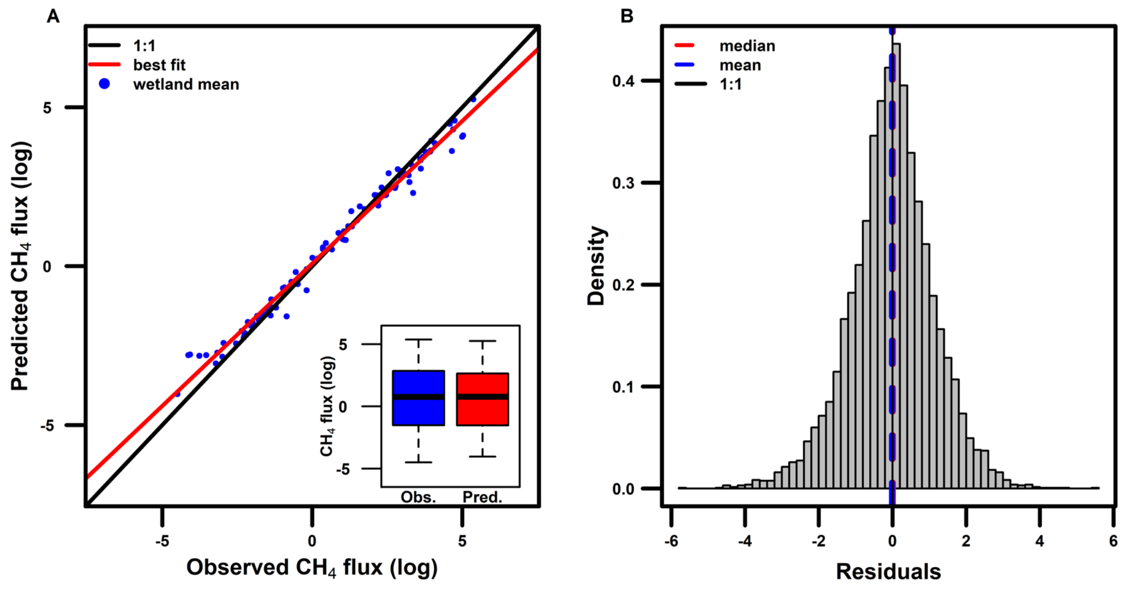

Fig. S4. (A) Scatter plot of observed versus predicted methane (CH_4) flux and (B) histogram of residual values from the random forest model of wetland CH_4 flux from the Prairie Pothole Region. (**A**) Solid black line indicates 1:1, red line indicates best fit, and blue dots represent wetland means; inset shows box and whisker plots of observed (Obs., blue) and predicted (Pred., red) CH_4 fluxes. Box plots depict the minimum, first quartile, median, third quartile, and maximum values. (**B**) Red and blue dashed lines indicate median and mean of residuals, respectively. Solid black line indicates residual of zero (i.e., no difference between observed and predicted, 1:1); all three lines in (**B**) overlap.

Table S1. Values of Q_{10} coefficients calculated for wetland methane (CH_4) and carbon dioxide (CO_2) fluxes. Values of Q_{10} were calculated using field-measured soil and air temperatures for the Prairie Pothole Region of North America. Values of Q_{10} were calculated over all temperatures and for each 10 °C increment (All, 0–10, 10–20, 20–30 °C) using data across all hydrologic conditions (All) as well as a subset of measurements in which soils were saturated (i.e., water-filled pore space was 100%). Soil temperatures were measured using probes inserted to 10 cm depth.

Data		All				Saturate soils only			
Temp. range	Temp. type	All	0–10	10–20	20–30	All	0–10	10–20	20–30
Q_{10} CH_4	soil	2.80	2.92	3.15	4.05	2.40	2.10	3.35	2.96
Q_{10} CO_2	soil	1.58	2.24	3.17	0.40	1.43	2.20	2.44	0.85
Q_{10} CH_4: CO_2	soil	1.77	1.30	0.99	10.21	1.68	0.95	1.37	3.48
Q_{10} CH_4	air	2.03	5.14	2.80	1.27	1.79	4.14	2.78	1.42
Q_{10} CO_2	air	2.35	5.99	2.25	1.69	1.85	8.59	1.86	1.37
Q_{10} CH_4: CO_2	air	0.86	0.86	1.25	0.75	0.96	0.48	1.50	1.03

Table S2. Wetland annual methane (CH_4) emissions estimates (Tg) from bottom-up and top-down global models for the year 2011 for the Prairie Pothole Region (PPR) of North America.

Model type	Bottom-up				Top-down	
Model source	Global Carbon Project v2		WetCHARTs (WC) v1.3.1		Global Carbon Project v2	
Model	Wetland map	PPR CH_4 (Tg)	Model	PPR CH_4 (Tg)	Model	PPR CH_4 (Tg)
CLASS-CTEM	WAD2M	0.587	WC-1913	1.817	CTE_GOSAT	1.953
DLEM	WAD2M	0.698	WC-1914	0.269	CTE_SURF	1.838
ELM	WAD2M	0.452	WC-1923	1.144	GELCA_SURF	0.461
JSBACH	WAD2M	0.210	WC-1924	0.165	LMDzPYVAR_GOSAT1	1.374
JULES	WAD2M	0.527	WC-1933	0.874	LMDzPYVAR_GOSAT2	0.840
LPJ-GUESS	WAD2M	0.668	WC-1934	0.125	LMDzPYVAR_GOSAT3	0.804
LPJ-MPI	WAD2M	0.254	WC-2913	2.423	LMDzPYVAR_GOSAT4	0.736
LPJ-WSL	WAD2M	0.242	WC-2914	0.359	LMDzPYVAR_GOSAT5	0.818
LPX	WAD2M	0.292	WC-2923	1.525	LMDzPYVAR_GOSAT6	0.711
ORCHIDEE	WAD2M	0.783	WC-2924	0.220	LMDzPYVAR_SURF1	1.097
TEM-MDM	WAD2M	2.262	WC-2933	1.165	LMDzPYVAR_SURF2	1.039
TRIPLEX-GHG	WAD2M	0.478	WC-2934	0.167	MIROCv4_SURF	0.784
VISIT	WAD2M	0.581	WC-3913	3.029	NICAM_SURF	0.935
**median**	**WAD2M**	**0.527**	WC-3914	0.449	NTF-4DVAR_NIES_GOSAT	0.966
**mean**	**WAD2M**	**0.618**	WC-3923	1.907	NTF-4DVAR_NIES_SURF	0.676
CLASS-CTEM	model	0.584	WC-3924	0.275	TM5-4DVAR_GOSAT1	0.835
ELM	model	0.359	WC-3933	1.456	TM5-4DVAR_GOSAT2	1.438
JSBACH	model	0.004	WC-3934	0.209	TM5-4DVAR_SURF1	0.619
JULES	model	0.239	**median**	**0.661**	TM5-4DVAR_SURF2	1.202
LPJ-MPI	model	1.518	**mean**	**0.977**	TM5-CAMS_GOSAT	1.173
LPJ-WSL	model	1.168			TM5-CAMS_SURF	1.155
LPX	model	0.209			**median**	**0.935**
ORCHIDEE	model	1.819			**mean**	**1.022**
VISIT	model	1.502				
**median**	**model**	**0.584**				
**mean**	**model**	**0.822**				

Bottom-up models include 13 biogeochemical models used in the Global Carbon Project version 2 (GCPv2), 18 models from WetCHARTs (WC) v1.3.1, and 21 top-down atmospheric inversion models used in GCPv2 that had estimates for 2011. For the bottom-up GCPv2 models, CH_4 estimates were calculated using two different wetland maps: the Wetland Area and Dynamics for Methane Modeling (WAD2M) and model-specific (model) wetland maps when available. The four-digit identifier for each WC model represents different model configurations based on terrestrial biosphere models, wetland extent scenarios, and CH_4: CO_2 temperature dependencies. Data for GCPv2 models obtained from Saunois et al. (*52*) and for WetCHARTs from Bloom et al. (*83*).

Table S3. Mean wetland methane (CH_4) emissions (Gg), flux (mg m^{-2} hr^{-1}), and environmental predictors by location and historical climate condition or future climate scenarios for the Prairie Pothole Region of North America.

Country	State/Prov	Year	Scenario	Condition	Annual CH_4 emissions (Gg)	CH_4 flux rate (mg m^{-2} hr^{-1})	Growing season (days)	Inundated area (km^2)	Wetland count	NDVI	Soil T (°C)	Hydroperiod (days)
CAN	AB	1991	Historical	Dry	38.7	0.48	266	6,131	610,099	0.17	9.8	149
CAN	AB	2011	Historical	Wet	56.8	0.78	210	7,814	794,669	0.24	11.3	127
CAN	AB	2100	SSP2-4.5	Dry	71.8(30)	0.90(29)	280(7)	6,130	610,099	0.13(15)	12.2(7)	150(8)
CAN	AB	2100	SSP2-4.5	Wet	137.9(28)	1.65(27)	280(7)	7,813	794,669	0.17(12)	12.1(7)	178(8)
CAN	AB	2100	SSP5-8.5	Dry	122.7(21)	1.39(17)	321(9)	6,130	610,099	0.11(16)	13.0(9)	176(9)
CAN	AB	2100	SSP5-8.5	Wet	227.3(21)	2.41(17)	321(9)	7,813	794,669	0.14(16)	13.0(9)	208(9)
CAN	MB	1991	Historical	Dry	26.4	0.82	210	1,894	369,529	0.27	12.6	89
CAN	MB	2011	Historical	Wet	31.4	1.18	210	3,108	340,932	0.25	12.8	112
CAN	MB	2100	SSP2-4.5	Dry	67.3(25)	2.10(24)	233(4)	1,893	369,529	0.25(5)	14.8(6)	94(4)
CAN	MB	2100	SSP2-4.5	Wet	72.1(19)	2.65(18)	233(4)	3,108	340,932	0.22(4)	14.9(6)	126(5)
CAN	MB	2100	SSP5-8.5	Dry	96.7(11)	2.73(6)	263(11)	1,893	369,529	0.21(16)	16.0(5)	108(13)
CAN	MB	2100	SSP5-8.5	Wet	98.1(13)	3.28(6)	263(11)	3,108	340,932	0.19(14)	16.2(5)	147(14)
CAN	SK	1991	Historical	Dry	107.8	0.58	210	14,917	1,590,566	0.19	12.1	110
CAN	SK	2011	Historical	Wet	171.7	0.83	210	23,509	1,811,345	0.19	12.1	127
CAN	SK	2100	SSP2-4.5	Dry	251.2(26)	1.32(24)	249(6)	14,911	1,590,566	0.16(7)	13.8(6)	133(6)
CAN	SK	2100	SSP2-4.5	Wet	408.5(22)	1.92(21)	249(6)	23,499	1,811,345	0.16(6)	13.9(6)	153(5)
CAN	SK	2100	SSP5-8.5	Dry	382.9(15)	1.80(9)	280(11)	14,911	1,590,566	0.13(22)	14.9(7)	156(12)
CAN	SK	2100	SSP5-8.5	Wet	584.1(13)	2.51(8)	280(11)	23,499	1,811,345	0.13(21)	15.0(6)	175(11)
USA	IA	1991	Historical	Dry	57.3	2.87	252	1,484	266,475	0.22	14.3	148
USA	IA	2011	Historical	Wet	10.9	1.50	280	300	58,686	0.19	13.3	137
USA	IA	2100	SSP2-4.5	Dry	90.3(7)	3.82(7)	323(6)	1,484	266,475	0.16	15.1(4)	200(8)
USA	IA	2100	SSP2-4.5	Wet	17.8(7)	2.15(4)	323(6)	300	58,686	0.17(3)	15.5(4)	156(7)
USA	IA	2100	SSP5-8.5	Dry	101.8(8)	4.16(8)	336(6)	1,484	266,475	0.16	16.9(8)	210(7)
USA	IA	2100	SSP5-8.5	Wet	19.0(6)	2.21(4)	336(6)	300	58,686	0.16(3)	17.3(8)	164(7)
USA	MN	1991	Historical	Dry	59.0	1.48	224	3,052	335,535	0.18	14.6	172
USA	MN	2011	Historical	Wet	121.6	1.79	238	5,601	617,752	0.20	13.7	149
USA	MN	2100	SSP2-4.5	Dry	102.9(10)	2.36(7)	284(7)	3,052	335,535	0.15(11)	15.4(5)	213(9)
USA	MN	2100	SSP2-4.5	Wet	209.5(7)	2.65(6)	284(7)	5,601	617,752	0.16(16)	15.4(5)	183(8)
USA	MN	2100	SSP5-8.5	Dry	115.7(7)	2.44(8)	314(10)	3,052	335,535	0.13(11)	16.6(7)	238(11)
USA	MN	2100	SSP5-8.5	Wet	234.3(10)	2.71(6)	314(10)	5,601	617,752	0.14(12)	16.7(7)	206(12)
USA	MT	1991	Historical	Dry	3.7	0.56	266	408	74,525	0.11	11.1	133
USA	MT	2011	Historical	Wet	5.8	1.14	224	675	90,681	0.17	12.1	124
USA	MT	2100	SSP2-4.5	Dry	8.6(23)	1.26(19)	282(8)	408	74,525	0.11(9)	13.5(6)	141(10)
USA	MT	2100	SSP2-4.5	Wet	16.0(19)	2.66(15)	282(8)	675	90,681	0.13(13)	13.5(6)	160(9)
USA	MT	2100	SSP5-8.5	Dry	12.9(16)	1.70(11)	323(8)	408	74,525	0.09(18)	14.4(8)	166(10)
USA	MT	2100	SSP5-8.5	Wet	23.3(15)	3.47(10)	323(8)	675	90,681	0.10(19)	14.4(8)	190(10)
USA	ND	1991	Historical	Dry	32.2	0.86	224	2,166	358,691	0.21	13.3	111
USA	ND	2011	Historical	Wet	140.7	1.47	210	11,670	783,018	0.22	13.6	141
USA	ND	2100	SSP2-4.5	Dry	71.1(13)	1.83(11)	257(6)	2,166	358,691	0.20(6)	15(5)	125(6)
USA	ND	2100	SSP2-4.5	Wet	273(11)	2.58(9)	257(6)	11,669	783,018	0.17(7)	15.1(5)	164(5)
USA	ND	2100	SSP5-8.5	Dry	88(10)	2.05(6)	290(8)	2,166	358,691	0.18(7)	16.2(7)	143(9)

USA	ND	2100	SSP5-8.5	Wet	331.3(9)	2.85(6)	290(8)	11,669	783,018	0.14(14)	16.3(7)	183(7)
USA	SD	1991	Historical	Dry	26.6	1.50	266	1,780	173,311	0.21	13.0	137
USA	SD	2011	Historical	Wet	81.8	2.10	252	3,548	295,866	0.20	13.1	156
USA	SD	2100	SSP2-4.5	Dry	47.3(7)	2.37(6)	314(6)	1,780	173,311	0.19(7)	14.5(5)	157(6)
USA	SD	2100	SSP2-4.5	Wet	136.1(8)	2.90(6)	314(6)	3,548	295,866	0.14(13)	14.6(5)	200(8)
USA	SD	2100	SSP5-8.5	Dry	50.9(7)	2.42(5)	333(6)	1,780	173,311	0.17(9)	16.2(7)	166(6)
USA	SD	2100	SSP5-8.5	Wet	152.2(8)	3.07(5)	333(6)	3,548	295,866	0.12(15)	16.2(7)	216(8)
CAN	All	1991	Historical	Dry	172.9	0.57	266	22,941	2,570,191	0.19	11.4	120
CAN	All	2011	Historical	Wet	259.9	0.85	210	34,431	2,946,946	0.22	12.0	126
CAN	All	2100	SSP2-4.5	Dry	390.3(26)	1.27(25)	280(7)	22,934	2,570,191	0.16(8)	13.4(6)	134(6)
CAN	All	2100	SSP2-4.5	Wet	618.5(23)	1.91(22)	280(7)	34,419	2,946,946	0.17(7)	13.4(6)	158(6)
CAN	All	2100	SSP5-8.5	Dry	602.3(16)	1.77(10)	321(9)	22,934	2,570,191	0.13(18)	14.4(7)	157(11)
CAN	All	2100	SSP5-8.5	Wet	909.5(15)	2.57(10)	321(9)	34,419	2,946,946	0.14(17)	14.4(7)	183(10)
USA	All	1991	Historical	Dry	178.9	1.33	266	8,890	1,208,537	0.20	13.5	137
USA	All	2011	Historical	Wet	360.8	1.67	280	21,794	1,846,003	0.20	13.4	145
USA	All	2100	SSP2-4.5	Dry	320.1(10)	2.24(8)	323(6)	8,889	1,208,537	0.17(6)	14.9(4)	162(7)
USA	All	2100	SSP2-4.5	Wet	652.4(9)	2.63(7)	323(6)	21,793	1,846,003	0.16(10)	15.0(4)	176(6)
USA	All	2100	SSP5-8.5	Dry	369.3(8)	2.41(6)	337(5)	8,889	1,208,537	0.16(8)	16.3(7)	180(8)
USA	All	2100	SSP5-8.5	Wet	760.1(9)	2.84(5)	337(5)	21,793	1,846,003	0.14(12)	16.4(7)	195(9)
PPR	All	1991	Historical	Dry	351.8	0.85	266	31,832	3,778,728	0.19	12.2	126
PPR	All	2011	Historical	Wet	620.7	1.16	280	56,225	4,792,949	0.21	12.5	133
PPR	All	2100	SSP2-4.5	Dry	710.4(19)	1.63(16)	323(6)	31,823	3,778,728	0.17(7)	13.9(5)	144(6)
PPR	All	2100	SSP2-4.5	Wet	1,270.9(16)	2.18(15)	323(6)	56,212	4,792,949	0.16(8)	14.0(5)	165(6)
PPR	All	2100	SSP5-8.5	Dry	971.5(13)	2.01(8)	337(5)	31,823	3,778,728	0.14(13)	15.1(7)	165(10)
PPR	All	2100	SSP5-8.5	Wet	1,669.6(12)	2.67(8)	337(5)	56,212	4,792,949	0.14(15)	15.1(7)	187(9)

Abbreviations: Prov-Province, CAN-Canada, PPR-Prairie Pothole Region*, AB-Alberta, MB-Manitoba, SK-Saskatchewan, IA-Iowa, MN-Minnesota, MT-Montana, ND-North Dakota, SD-South Dakota, SSP-Socioeconomic Pathway, NDVI-Normalized Difference Vegetation Index, Soil T-soil temperature. *PPR aggregates data from USA and CAN. For CH_4 flux rates, the values in parentheses represent the coefficient of variation (CV, expressed as a percent) around the prediction means of 13 Earth Systems Models from the Coupled Model Intercomparison Project Phase 6. For environmental predictors, values in parentheses represent the CV of values used in the models. Note that values from historical conditions do not have CV values because they were not based on multiple models.

REFERENCES AND NOTES

1. B. Poulter, P. Bousquet, J. G. Canadell, P. Ciais, A. Peregon, M. Saunois, V. K. Arora, D. J. Beerling, V. Brovkin, C. D. Jones, F. Joos, N. Gedney, A. Ito, T. Kleinen, C. D. Koven, K. McDonald, J. R. Melton, C. Peng, S. Peng, C. Prigent, R. Schroeder, W. J. Riley, M. Saito, R. Spahni, H. Tian, L. Taylor, N. Viovy, D. Wilton, A. Wiltshire, X. Xu, B. Zhang, Z. Zhang, Q. Zhu, Global wetland contribution to 2000–2012 atmospheric methane growth rate dynamics. *Environ. Res. Lett.* **12**, 094013 (2017).

2. M. Saunois, A. R. Stavert, B. Poulter, P. Bousquet, J. G. Canadell, R. B. Jackson, P. A. Raymond, E. J. Dlugokencky, S. Houweling, P. K. Patra, P. Ciais, V. K. Arora, D. Bastviken, P. Bergamaschi, D. R. Blake, G. Brailsford, L. Bruhwiler, K. M. Carlson, M. Carrol, S. Castaldi, N. Chandra, C. Crevoisier, P. M. Crill, K. Covey, C. L. Curry, G. Etiope, C. Frankenberg, N. Gedney, M. I. Hegglin, L. Höglund-Isaksson, G. Hugelius, M. Ishizawa, A. Ito, G. Janssens-Maenhout, K. M. Jensen, F. Joos, T. Kleinen, P. B. Krummel, R. L. Langenfelds, G. G. Laruelle, L. Liu, T. Machida, S. Maksyutov, K. C. McDonald, J. McNorton, P. A. Miller, J. R. Melton, I. Morino, J. Müller, F. Murguia-Flores, V. Naik, Y. Niwa, S. Noce, S. O'Doherty, R. J. Parker, C. Peng, S. Peng, G. P. Peters, C. Prigent, R. Prinn, M. Ramonet, P. Regnier, W. J. Riley, J. A. Rosentreter, A. Segers, I. J. Simpson, H. Shi, S. J. Smith, L. P. Steele, B. F. Thornton, H. Tian, Y. Tohjima, F. N. Tubiello, A. Tsuruta, N. Viovy, A. Voulgarakis, T. S. Weber, M. van Weele, G. R. van der Werf, R. F. Weiss, D. Worthy, D. Wunch, Y. Yin, Y. Yoshida, W. Zhang, Z. Zhang, Y. Zhao, B. Zheng, Q. Zhu, Q. Zhu, Q. Zhuang, The global methane budget 2000–2017. *Earth Syst. Sci. Data* **12**, 1561–1623 (2020).

3. G. Myhre, D. Shindell, F.-M. Bréon, W. J. Collins, J. Fuglestvedt, J. Huang, D. Koch, J.-F. Lamarque, D. Lee, B. Mendoza, T. Nakajima, A. Robock, G. Stephens, T. Takemura, H. Zhang, Anthropogenic and natural radiative forcing, in *Climate Change 2013: The Physical Science Basis. Contribution of Working Group I to the Fifth Assessment Report of the Intergovernmental Panel on Climate Change*, T. F. Stocker, D. Qin, G.-K. Plattner, M. Tignor, S. K. Allen, J. Boschung, A. Nauels, Y. Xia, V. Bex, P. M. Midgley, Eds. (Cambridge Univ. Press, 2013), chap. 8.

4. E. G. Nisbet, R. E. Fisher, D. Lowry, J. L. France, G. Allen, S. Bakkaloglu, T. J. Broderick, M. Cain, M. Coleman, J. Fernandez, G. Forster, P. T. Griffiths, C. P. Iverach, B. F. J. Kelly, M. R. Manning, P. B. R. Nisbet-Jones, J. A. Pyle, A. Townsend-Small, A. Alshalaan, N. Warwick, G. Zazzeri, Methane mitigation: Methods to reduce emissions, on the path to the Paris Agreement. *Rev. Geophys.* **58**, e2019RG000675 (2020).

5. R. B. Jackson, S. Abernethy, J. G. Canadell, M. Cargnello, S. J. Davis, S. Féron, S. Fuss, A. J. Heyer, C. Hong, C. D. Jones, H. Damon Matthews, F. M. O'Connor, M. Pisciotta, H. M. Rhoda, R. de Richter, E. I. Solomon, J. L. Wilcox, K. Zickfeld, Atmospheric methane removal: A research agenda. *Philos. Trans. Royal Soc. A* **379**, 20200454 (2021).

6. European Commission and United States of America, Global methane pledge, www.ccacoalition.org/en/resources/global-methane-pledge (2021).

7. T. Lauvaux, C. Giron, M. Mazzolini, A. d'Aspremont, R. Duren, D. Cusworth, D. Shindell, P. Ciais, Global assessment of oil and gas methane ultra-emitters. *Science* **375**, 557–561 (2022).

8. J. A. Rosentreter, A. V. Borges, B. R. Deemer, M. A. Holgerson, S. Liu, C. Song, J. Melack, P. A. Raymond, C. M. Duarte, G. H. Allen, D. Olefeldt, B. Poulter, T. I. Battin, B. D. Eyre, Half of global methane emissions come from highly variable aquatic ecosystem sources. *Nat. Geosci.* **14**, 225–230 (2021).

9. S. D. Bridgham, H. Cadillo-Quiroz, J. K. Keller, Q. Zhuang, Methane emissions from wetlands: Biogeochemical, microbial, and modeling perspectives from local to global scales. *Glob. Chang. Biol.* **19**, 1325–1346 (2013).

10. G. Yvon-Durocher, A. P. Allen, D. Bastviken, R. Conrad, C. Gudasz, A. St-Pierre, N. Thanh-Duc, P. A. del Giorgio, Methane fluxes show consistent temperature dependence across microbial to ecosystem scales. *Nature* **507**, 488–491 (2014).

11. Z. Zhang, N. E. Zimmermann, A. Stenke, X. Li, E. L. Hodson, G. Zhu, C. Huang, B. Poulter, Emerging role of wetland methane emissions in driving 21st century climate change. *Proc. Natl. Acad. Sci. U.S.A.* **114**, 9647–9652 (2017).

12. A. R. Stavert, M. Saunois, J. G. Canadell, B. Poulter, R. B. Jackson, P. Regnier, R. Lauerwald, P. A. Raymond, G. H. Allen, P. K. Patra, P. Bergamaschi, P. Bousquet, N. Chandra, P. Ciais, A. Gustafson, M. Ishizawa, A. Ito, T. Kleinen, S. Maksyutov, J. McNorton, J. R. Melton, J. Müller, Y. Niwa, S. Peng, W. J. Riley, A. Segers, H. Tian, A. Tsuruta, Y. Yin, Z. Zhang, B. Zheng, Q. Zhuang, Regional trends and drivers of the global methane budget. *Glob. Chang. Biol.* **28**, 182–200 (2022).

13. D. A. Freedman, Ecological inference, in *International Encyclopedia of the Social & Behavioral Sciences*, N. J. Smelser, P. B. Baltes, Eds. (Pergamon, 2001), pp. 4027–4030.

14. A. A. Bloom, K. W. Bowman, M. Lee, A. J. Turner, R. Schroeder, J. R. Worden, R. Weidner, K. C. McDonald, D. J. Jacob, A global wetland methane emissions and uncertainty dataset for atmospheric chemical transport models (WetCHARTs version 1.0). *Geosci. Model Dev.* **10**, 2141–2156 (2017).

15. P. A. Keddy, L. H. Fraser, A. I. Solomeshch, W. J. Junk, D. R. Campbell, M. T. K. Arroyo, C. J. R. Alho, Wet and wonderful: The world's largest wetlands are conservation priorities. *Bioscience* **59**, 39–51 (2009).

16. T. E. Dahl, "Status and trends of prairie wetlands in the United States 1997 to 2009" (U.S. Fish and Wildlife Service, 2014)

17. M. B. Goldhaber, C. T. Mills, J. M. Morrison, C. A. Stricker, D. M. Mushet, J. W. LaBaugh, Hydrogeochemistry of prairie pothole region wetlands: Role of long-term critical zone processes. *Chem. Geol.* **387**, 170–183 (2014).

18. R. A. Gleason, N. H. Euliss, Jr., B. A. Tangen, M. K. Laubhan, B. A. Browne, USDA conservation program and practice effects on wetland ecosystem services in the Prairie Pothole Region. *Ecol. Appl.* **21**, S65–S81 (2011).

19. J. M. Marton, I. F. Creed, D. B. Lewis, C. R. Lane, N. B. Basu, M. J. Cohen, C. B. Craft, Geographically isolated wetlands are important biogeochemical reactors on the landscape. *Bioscience* **65**, 408–418 (2015).

20. B. A. Tangen, S. Bansal, Soil organic carbon stocks and sequestration rates of inland, freshwater wetlands: Sources of variability and uncertainty. *Sci. Total Environ.* **749**, 141444 (2020).

21. R. A. Gleason, B. A. Tangen, B. A. Browne, N. H. Euliss, Jr., Greenhouse gas flux from cropland and restored wetlands in the Prairie Pothole Region. *Soil Biol. Biochem.* **41**, 2501–2507 (2009).

22. B. A. Tangen, R. G. Finocchiaro, R. A. Gleason, Effects of land use on greenhouse gas fluxes and soil properties of wetland catchments in the Prairie Pothole Region of North America. *Sci. Total Environ.* **533**, 391–409 (2015).

23. D. Pennock, T. Yates, A. Bedard-Haughn, K. Phipps, R. Farrell, R. McDougal, Landscape controls on N_2O and CH_4 emissions from freshwater mineral soil wetlands of the Canadian Prairie Pothole region. *Geoderma* **155**, 308–319 (2010).

24. P. Dalcin Martins, D. W. Hoyt, S. Bansal, C. T. Mills, M. Tfaily, B. A. Tangen, R. G. Finocchiaro, M. D. Johnston, B. C. McAdams, M. J. Solensky, G. J. Smith, Y.-P. Chin, M. J. Wilkins, Abundant carbon substrates drive extremely high sulfate reduction rates and methane fluxes in Prairie Pothole Wetlands. *Glob. Chang. Biol.* **23**, 3107–3120 (2017).

25. P. Badiou, R. McDougal, D. Pennock, B. Clark, Greenhouse gas emissions and carbon sequestration potential in restored wetlands of the Canadian prairie pothole region. *Wetl. Ecol. Manag.* **19**, 237–256 (2011).

26. M. A. Holgerson, P. A. Raymond, Large contribution to inland water CO_2 and CH_4 emissions from very small ponds. *Nat. Geosci.* **9**, 222–226 (2016).

27. I. F. Creed, J. Miller, D. Aldred, J. K. Adams, S. Spitale, R. A. Bourbonniere, Hydrologic profiling for greenhouse gas effluxes from natural grasslands in the prairie pothole region of Canada. *Eur. J. Vasc. Endovasc. Surg.* **118**, 680–697 (2013).

28. S. H. Knox, S. Bansal, G. McNicol, K. Schafer, C. Sturtevant, M. Ueyama, A. C. Valach, D. Baldocchi, K. Delwiche, A. R. Desai, E. Euskirchen, J. Liu, A. Lohila, A. Malhotra, L. Melling, W. Riley, B. R. K. Runkle, J. Turner, R. Vargas, Q. Zhu, T. Alto, E. Fluet-

Chouinard, M. Goeckede, J. R. Melton, O. Sonnentag, T. Vesala, E. Ward, Z. Zhang, *S. Feron*, Z. Ouyang, P. Alekseychik, M. Aurela, G. Bohrer, D. I. Campbell, J. Chen, H. Chu, H. J. Dalmagro, J. P. Goodrich, P. Gottschalk, T. Hirano, H. Iwata, G. Jurasinski, M. Kang, F. Koebsch, I. Mammarella, M. B. Nilsson, K. Ono, M. Peichl, O. Peltola, Y. Ryu, T. Sachs, A. Sakabe, J. P. Sparks, E.-S. Tuittila, G. L. Vourlitis, G. X. Wong, L. Windham-Myers, B. Poulter, R. B. Jackson, Identifying dominant environmental predictors of freshwater wetland methane fluxes across diurnal to seasonal time scales. *Glob. Chang. Biol.* **27**, 3582–3604 (2021).

29. M. R. Turetsky, A. Kotowska, J. Bubier, N. B. Dise, P. Crill, E. R. C. Hornibrook, K. Minkkinen, T. R. Moore, I. H. Myers-Smith, H. Nykänen, D. Olefeldt, J. Rinne, S. Saarnio, N. Shurpali, E.-S. Tuittila, J. M. Waddington, J. R. White, K. P. Wickland, M. Wilmking, A synthesis of methane emissions from 71 northern, temperate, and subtropical wetlands. *Glob. Chang. Biol.* **20**, 2183–2197 (2014).

30. J. Le Mer, P. Roger, Production, oxidation, emission and consumption of methane by soils: A review. *Eur. J. Soil Biol.* **37**, 25–50 (2001).

31. P. Roslev, G. M. King, Regulation of methane oxidation in a freshwater wetland by water table changes and anoxia. *FEMS Microbiol. Ecol.* **19**, 105–115 (1996).

32. J. R. Melton, R. Wania, E. L. Hodson, B. Poulter, B. Ringeval, R. Spahni, T. Bohn, C. A. Avis, D. J. Beerling, G. Chen, A. V. Eliseev, S. N. Denisov, P. O. Hopcroft, D. P. Lettenmaier, W. J. Riley, J. S. Singarayer, Z. M. Subin, H. Tian, S. Zürcher, V. Brovkin, P. M. van Bodegom, T. Kleinen, Z. C. Yu, J. O. Kaplan, Present state of global wetland extent and wetland methane modelling: Conclusions from a model inter-comparison project (WETCHIMP). *Biogeosciences* **10**, 753–788 (2013).

33. T. R. Christensen, A. Ekberg, L. Ström, M. Mastepanov, N. Panikov, M. Öquist, B. H. Svensson, H. Nykänen, P. J. Martikainen, H. Oskarsson, Factors controlling large scale variations in methane emissions from wetlands. *Geophys. Res. Lett.* **30**, 1414–1419 (2003).

34. H. J. Laanbroek, Methane emission from natural wetlands: Interplay between emergent macrophytes and soil microbial processes. A mini-review. *Ann. Bot.* **105**, 141–153 (2010).

35. S. Bansal, O. F. Johnson, J. Meier, X. Zhu, Vegetation affects timing and location of wetland methane emissions. *J. Geophys. Res. Biogeosci.* **125**, e2020JG005777 (2020).

36. B. A. Tangen, S. Bansal, J. R. Freeland, S. E. Travis, J. D. Wasko, T. P. McGonigle, L. G. Goldsborough, K. Gow, J. E. Marburger, J. A. Meier, Distributions of native and invasive *Typha* (cattail) throughout the Prairie Pothole Region of North America. *Wetl. Ecol. Manag.* **30**, 1–17 (2022).

37. S. Bansal, S. C. Lishawa, S. Newman, B. A. Tangen, D. Wilcox, D. Albert, M. J. Anteau, M. J. Chimney, R. L. Cressey, E. DeKeyser, K. J. Elgersma, S. A. Finkelstein, J. Freeland, R. Grosshans, P. E. Klug, D. J. Larkin, B. A. Lawrence, G. Linz, J. Marburger, G. Noe, *C. Otto*, N. Reo, J. Richards, C. Richardson, L. Rodgers, A. J. Schrank, D. Svedarsky, S. Travis, N. Tuchman, L. Windham-Myers, *Typha* (Cattail) invasion in North American wetlands: Biology, regional problems, impacts, ecosystem services, and management. *Wetlands* **39**, 645–684 (2019).

38. J. J. Beaulieu, T. DelSontro, J. A. Downing, Eutrophication will increase methane emissions from lakes and impoundments during the 21st century. *Nat. Commun.* **10**, 1375 (2019).

39. S. Bansal, B. A. Tangen, R. A. Gleason, P. Badiou, I. F. Creed, Land management strategies influence soil organic carbon stocks of prairie potholes of North America, in *Wetland Carbon and Environmental Management*, K. W. Krauss, Z. Zhu, C. L. Stagg, Eds. (AGU and Wiley, ed. 1, 2022), pp. 273–285.

40. T. Sohl, J. Dornbierer, S. Wika, C. Robison, Remote sensing as the foundation for high-resolution United States landscape projections – The Land Change Monitoring, assessment, and projection (LCMAP) initiative. *Environ. Model. Software* **120**, 104495 (2019).

41. D. Bastviken, J. Cole, M. Pace, L. Tranvik, Methane emissions from lakes: Dependence of lake characteristics, two regional assessments, and a global estimate. *Global Biogeochem. Cy.* **18**, GB4009 (2004).

42. T. DelSontro, J. J. Beaulieu, J. A. Downing, Greenhouse gas emissions from lakes and impoundments: Upscaling in the face of global change. *Limnol. Oceanogr. Lett.* **3**, 64–75 (2018).

43. J. Kiss, A. Bedard-Haughn, Predictive mapping of solute-rich wetlands in the Canadian Prairie Pothole Region through high-resolution digital elevation model analyses. *Wetlands* **41**, 38 (2021).

44. N. H. Euliss, Jr., J. W. LaBaugh, L. H. Fredrickson, D. M. Mushet, M. K. Laubhan, G. A. Swanson, T. C. Winter, D. O. Rosenberry, R. D. Nelson, The wetland continuum: A conceptual framework for interpreting biological studies. *Wetlands* **24**, 448–458 (2004).

45. O. P. McKenna, D. M. Mushet, D. O. Rosenberry, J. W. LaBaugh, Evidence for a climate-induced ecohydrological state shift in wetland ecosystems of the southern Prairie Pothole Region. *Clim. Change* **145**, 273–287 (2017).

46. M. K. Vanderhoof, L. C. Alexander, The role of lake expansion in altering the wetland landscape of the Prairie Pothole Region, United States, United States. *Wetlands* **36**, 309–321 (2016).

47. J. W. Jones, Improved automated detection of subpixel-scale inundation—Revised Dynamic Surface Water Extent (DSWE) partial surface water tests. *Remote Sens. (Basel)* **11**, 374 (2019).

48. M. Mastepanov, C. Sigsgaard, E. J. Dlugokencky, S. Houweling, L. Ström, M. P. Tamstorf, T. R. Christensen, Large tundra methane burst during onset of freezing. *Nature* **456**, 628–630 (2008).

49. D. Zona, B. Gioli, R. Commane, J. Lindaas, S. C. Wofsy, C. E. Miller, S. J. Dinardo, S. Dengel, C. Sweeney, A. Karion, R. Y.-W. Chang, J. M. Henderson, P. C. Murphy, J. P. Goodrich, V. Moreaux, A. Liljedahl, J. D. Watts, J. S. Kimball, D. A. Lipson, W. C. Oechel, Cold season emissions dominate the Arctic tundra methane budget. *Proc. Natl. Acad. Sci. U.S.A.* **113**, 40–45 (2016).

50. K. B. Delwiche, S. H. Knox, A. Malhotra, E. Fluet-Chouinard, G. McNicol, *S. Feron*, Z. Ouyang, D. Papale, C. Trotta, E. Canfora, Y. W. Cheah, D. Christianson, M. C. R. Alberto, P. Alekseychik, M. Aurela, D. Baldocchi, S. Bansal, D. P. Billesbach, G. Bohrer, R. Bracho, N. Buchmann, D. I. Campbell, G. Celis, J. Chen, W. Chen, H. Chu, H. J. Dalmagro, S. Dengel, A. R. Desai, M. Detto, H. Dolman, E. Eichelmann, E. Euskirchen, D. Famulari, K.

Fuchs, M. Goeckede, S. Gogo, M. J. Gondwe, J. P. Goodrich, P. Gottschalk, S. L. Graham, M. Heimann, M. Helbig, C. Helfter, K. S. Hemes, T. Hirano, D. Hollinger, L. Hörtnagl, H. Iwata, A. Jacotot, G. Jurasinski, M. Kang, K. Kasak, J. King, J. Klatt, F. Koebsch, K. W. Krauss, D. Y. F. Lai, A. Lohila, I. Mammarella, L. Belelli Marchesini, *G. Manca*, J. H. Matthes, T. Maximov, L. Merbold, B. Mitra, T. H. Morin, E. Nemitz, M. B. Nilsson, S. Niu, W. C. Oechel, P. Y. Oikawa, K. Ono, M. Peichl, O. Peltola, M. L. Reba, A. D. Richardson, W. Riley, B. R. K. Runkle, Y. Ryu, T. Sachs, A. Sakabe, C. R. Sanchez, E. A. Schuur, K. V. R. Schäfer, O. Sonnentag, J. P. Sparks, E. Stuart-Haëntjens, C. Sturtevant, R. C. Sullivan, D. J. Szutu, J. E. Thom, M. S. Torn, E. S. Tuittila, J. Turner, M. Ueyama, A. C. Valach, R. Vargas, A. Varlagin, A. Vazquez-Lule, J. G. Verfaillie, T. Vesala, G. L. Vourlitis, E. J. Ward, C. Wille, G. Wohlfahrt, G. X. Wong, Z. Zhang, D. Zona, L. Windham-Myers, B. Poulter, R. B. Jackson, FLUXNET-CH4: A global, multi-ecosystem dataset and analysis of methane seasonality from freshwater wetlands. *Earth Syst. Sci. Data* **13**, 3607–3689 (2021).

51. M. Jammet, P. Crill, S. Dengel, T. Friborg, Large methane emissions from a subarctic lake during spring thaw: Mechanisms and landscape significance. *Eur. J. Vasc. Endovasc. Surg.* **120**, 2289–2305 (2015).

52. M. Saunois, A. R. Stavert, B. Poulter, P. Bousquet, J. G. Canadell, R. B. Jackson, P. A. Raymond, E. J. Dlugokencky, S. Houweling, P. K. Patra, P. Ciais, V. K. Arora, D. Bastviken, P. Bergamaschi, D. R. Blake, G. Brailsford, L. Bruhwiler, K. M. Carlson, M. Carrol, S. Castaldi, N. Chandra, C. Crevoisier, P. M. Crill, K. Covey, C. L. Curry, G. Etiope, C. Frankenberg, N. Gedney, M. I. Hegglin, L. Höglund-Isaksson, G. Hugelius, M. Ishizawa, A. Ito, G. Janssens-Maenhout, K. M. Jensen, F. Joos, T. Kleinen, P. B. Krummel, R. L. Langenfelds, G. G. Laruelle, L. Liu, T. Machida, S. Maksyutov, K. C. McDonald, J. McNorton, P. A. Miller, J. R. Melton, I. Morino, J. Müller, F. Murguia-Flores, V. Naik, Y. Niwa, S. Noce, S. O'Doherty, R. J. Parker, C. Peng, S. Peng, G. P. Peters, C. Prigent, R. Prinn, M. Ramonet, P. Regnier, W. J. Riley, J. A. Rosentreter, A. Segers, I. J. Simpson, H. Shi, S. J. Smith, L. P. Steele, B. F. Thornton, H. Tian, Y. Tohjima, F. N. Tubiello, A. Tsuruta, N. Viovy, A. Voulgarakis, T. S. Weber, M. van Weele, G. R. van der Werf, R. F. Weiss, D. Worthy, D. Wunch, Y. Yin, Y. Yoshida, W. Zhang, Z. Zhang, Y. Zhao, B. Zheng,

Q. Zhu, Q. Zhu, Q. Zhuang, "Supplemental data of the Global Carbon Project Methane Budget 2019 (Version 2.0)" (Data set, Global Carbon Project, 2020)

53. Z. Zhang, E. Fluet-Chouinard, K. Jensen, K. McDonald, G. Hugelius, T. Gumbricht, M. Carroll, C. Prigent, A. Bartsch, B. Poulter, Development of the global dataset of Wetland Area and Dynamics for Methane Modeling (WAD2M). *Earth Syst. Sci. Data* **13**, 2001–2023 (2021).

54. V. Eyring, S. Bony, G. A. Meehl, C. A. Senior, B. Stevens, R. J. Stouffer, K. E. Taylor, Overview of the Coupled Model Intercomparison Project Phase 6 (CMIP6) experimental design and organization. *Geosci. Model Dev.* **9**, 1937–1958 (2016).

55. O. P. McKenna, D. M. Mushet, S. R. Kucia, E. C. McCulloch-Huseby, Limited shifts in the distribution of migratory bird breeding habitat density in response to future changes in climate. *Ecol. Appl.* **31**, e02428 (2021).

56. I. F. Creed, C. R. Lane, J. N. Serran, L. C. Alexander, N. B. Basu, A. J. K. Calhoun, J. R. Christensen, M. J. Cohen, C. Craft, E. D'Amico, E. DeKeyser, L. Fowler, H. E. Golden, J. W. Jawitz, P. Kalla, L. K. Kirkman, M. Lang, S. G. Leibowitz, D. B. Lewis, J. Marton, D. L. McLaughlin, H. Raanan-Kiperwas, M. C. Rains, K. C. Rains, L. Smith, Enhancing protection for vulnerable waters. *Nat. Geosci.* **10**, 809–815 (2017).

57. R. Kolka, C. Trettin, W. Tang, K. Krauss, S. Bansal, J. Drexler, K. Wickland, R. Chimner, D. Hogan, E. J. Pindilli, B. Benscoter, B. Tangen, E. Kane, S. Bridgham, C. Richardson "Terrestrial wetlands," in *Second State of the Carbon Cycle Report (SOCCR2): A Sustained Assessment Report*, N. Cavallaro, G. Shrestha, R. Birdsey, M. A. Mayes, R. G. Najjar, S. C. Reed, P. Romero-Lankao, Z. Zhu, Eds. (U.S. Global Change Research Program, 2018), chap. 13.

58. IPCC, *Climate Change 2013: The Physical Science Basis. Contribution of Working Group I to the Fifth Assessment Report of the Intergovernmental Panel on Climate Change*, T. F. Stocker, D. Qin, G.-K. Plattner, M. Tignor, S. K. Allen, J. Boschung, A. Nauels, Y. Xia, V. Bex, P. M. Midgley, Eds. (Cambridge Univ. Press, 2013).

59. EPA, "Global non-CO_2 greenhouse gas emission projections and mitigation 2015–2050" (EPA-430-R-19-010, U.S. Environmental Protection Agency, 2019); www.epa.gov/sites/default/files/2019-09/documents/epa_non-co2_greenhouse_gases_rpt-epa430r19010.pdf.

60. K. J. Van Meter, N. B. Basu, Signatures of human impact: Size distributions and spatial organization of wetlands in the Prairie Pothole landscape. *Ecol. Appl.* **25**, 451–465 (2015).

61. L. M. Cowardin, V. Carter, F. C. Golet, E. T. LaRoe, "Classification of wetlands and deepwater habitats of the United States" (FWS/OBS-79/31, U.S. Fish and Wildlife Service, 1979)

62. B. A. Tangen, R. G. Finocchiaro, R. A. Gleason, C. F. Dahl, Greenhouse gas fluxes of a shallow lake in south-central North Dakota, USA, *Wetlands* **36**, 779–787 (2016).

63. B. Millett, W. C. Johnson, G. Guntenspergen, Climate trends of the North American prairie pothole region 1906–2000. *Clim. Change* **93**, 243–267 (2009).

64. T. Wang, A. Hamann, D. Spittlehouse, C. Carroll, Locally downscaled and spatially customizable climate data for historical and future periods for North America. *PLOS ONE* **11**, e0156720 (2016).

65. P. E. Todhunter, R. Fietzek-DeVries, Natural hydroclimatic forcing of historical lake volume fluctuations at Devils Lake, North Dakota (USA). *Nat. Hazards* **81**, 1515–1532 (2016).

66. T. C. Winter, D. O. Rosenberry, The interaction of ground water with prairie pothole wetlands in the Cottonwood Lake area, east-central North Dakota, 1979–1990. *Wetlands* **15**, 193–211 (1995).

67. J. Zou, A. D. Ziegler, D. Chen, G. McNicol, P. Ciais, X. Jiang, C. Zheng, J. Wu, J. Wu, Z. Lin, X. He, L. E. Brown, J. Holden, Z. Zhang, S. J. Ramchunder, A. Chen, Z. Zeng, Rewetting global wetlands effectively reduces major greenhouse gas emissions. *Nat. Geosci.* **15**, 627–632 (2022).

68. R. Finocchiaro, B. Tangen, R. Gleason, Greenhouse gas fluxes of grazed and hayed wetland catchments in the U.S. Prairie Pothole Ecoregion. *Wetl. Ecol. Manag.* **22**, 305–324 (2014).

69. B. A. Tangen, S. Bansal, “Soil properties and greenhouse gas fluxes of Prairie Pothole Region wetlands: A comprehensive data release” (data release, U.S. Geological Survey, 2019); www.sciencebase.gov/catalog/item/59a86e39e4b0421949a84627.

70. S. Bansal, B. A. Tangen, “Methane flux model for wetlands of the Prairie Pothole Region of North America: Model input data and programming code” (data release, U.S. Geological Survey, 2022); www.sciencebase.gov/catalog/item/6227d1c5d34ee0c6b38b8051.

71. S. Bansal, B. Tangen, R. Finocchiaro, Diurnal patterns of methane flux from a seasonal wetland: Mechanisms and methodology. *Wetlands* **38**, 933–943 (2018).

72. S. N. Wood, *Generalized Additive Models: An Introduction with R* (Chapman and Hall/CRC, ed. 2, 2017).

73. K. L. Hondula, B. DeVries, C. N. Jones, M. A. Palmer, Effects of using high resolution satellite-based inundation time series to estimate methane fluxes from forested wetlands. *Geophys. Res. Lett.* **48**, 2021GL092556 (2021).

74. S. N. Wood, Fast stable restricted maximum likelihood and marginal likelihood estimation of semiparametric generalized linear models. *J. R. Stat. Soc. Ser. B Stat. Methodol.* **73**, 3–36 (2011).

75. M. A. Birk, respirometry: Tools for conducting and analyzing respirometry experiments. R package version 1.3.0 (2021); https://CRAN.R-project.org/package=respirometry.

76. C. R. Mahony, T. Wang, A. Hamann, A. J. Cannon, A global climate model ensemble for downscaled monthly climate normals over North America. *Int. J. Climatol.* **42**, 5871, 5891 (2022).

77. G. Tramontana, K. Ichii, G. Camps-Valls, E. Tomelleri, D. Papale, Uncertainty analysis of gross primary production upscaling using Random Forests, remote sensing and eddy covariance data. *Remote Sens. Environ.* **168**, 360–373 (2015).

78. P. Bodesheim, M. Jung, F. Gans, M. D. Mahecha, M. Reichstein, Upscaled diurnal cycles of land–atmosphere fluxes: A new global half-hourly data product. *Earth Syst. Sci. Data* **10**, 1327–1365 (2018).

79. A. Räsänen, T. Manninen, M. Korkiakoski, A. Lohila, T. Virtanen, Predicting catchment-scale methane fluxes with multi-source remote sensing. *Landsc. Ecol.* **36**, 1177–1195 (2021).

80. L. Breiman, Random Forests. *Mach. Learn.* **45**, 5–32 (2001).

81. J. Irvin, S. Zhou, G. McNicol, F. Lu, V. Liu, E. Fluet-Chouinard, Z. Ouyang, S. H. Knox, A. Lucas-Moffat, C. Trotta, D. Papale, D. Vitale, I. Mammarella, P. Alekseychik, M. Aurela, A. Avati, D. Baldocchi, S. Bansal, G. Bohrer, D. I. Campbell, J. Chen, H. Chu, H. J. Dalmagro, K. B. Delwiche, A. R. Desai, E. Euskirchen, *S. Feron*, M. Goeckede, M. Heimann, M. Helbig, C. Helfter, K. S. Hemes, T. Hirano, H. Iwata, G. Jurasinski, A. Kalhori, A. Kondrich, D. Y. F. Lai, A. Lohila, A. Malhotra, L. Merbold, B. Mitra, A. Ng, M. B. Nilsson, A. Noormets, M. Peichl, A. C. Rey-Sanchez, A. D. Richardson, B. R. K. Runkle, K. V. R. Schäfer, O. Sonnentag, E. Stuart-Haëntjens, C. Sturtevant, M. Ueyama, A. C. Valach, R. Vargas, G. L. Vourlitis, E. J. Ward, G. X. Wong, D. Zona, M. C. R. Alberto, D. P. Billesbach, G. Celis, H. Dolman, T. Friborg, K. Fuchs, S. Gogo, M. J. Gondwe, J. P. Goodrich, P. Gottschalk, L. Hörtnagl, A. Jacotot, F. Koebsch, K. Kasak, R. Maier, T. H. Morin, E. Nemitz, W. C. Oechel, P. Y. Oikawa, K. Ono, T. Sachs, A. Sakabe, E. A. Schuur, R. Shortt, R. C. Sullivan, D. J. Szutu, E.-S. Tuittila, A. Varlagin, J. G. Verfaillie, C. Wille, L. Windham-Myers, B. Poulter, R. B. Jackson, Gap-filling eddy covariance methane fluxes: Comparison of machine learning model predictions and uncertainties at FLUXNET-CH4 wetlands. *Agric. For. Meteorol.* **308–309**, 108528 (2021).

82. X. Yu, D. B. Millet, K. C. Wells, D. K. Henze, H. Cao, T. J. Griffis, E. A. Kort, G. Plant, M. J. Deventer, R. K. Kolka, D. T. Roman, K. J. Davis, A. R. Desai, B. C. Baier, K. McKain, A. C. Czarnetzki, A. A. Bloom, Aircraft-based inversions quantify the importance of wetlands and livestock for Upper Midwest methane emissions. *Atmos. Chem. Phys.* **21**, 951–971 (2021).

83. A. A. Bloom, K. W. Bowman, M. Lee, A. J. Turner, R. Schroeder, J. R. Worden, R. J. Weidner, K. C. McDonald, D. J. Jacob, CMS: Global 0.5-deg wetland methane emissions and uncertainty (WetCHARTs v1.3.1) (2021); https://daac.ornl.gov/cgi-bin/dsviewer.pl?ds_id=1915.

84. J. T. Falgout, J. Gordon, B. Williams, M. J. Davis, SGS Advanced Research Computing, USGS Denali Supercomputer, https://doi.org/10.5066/P9PSW367.

85. C. E. Soulard, J. J. Walker, R. E. Petrakis, Implementation of a surface water extent model in Cambodia using cloud-based remote sensing. *Remote Sens. (Basel)* **12**, 984 (2020).

86. J. C. Angle, T. H. Morin, L. M. Solden, A. B. Narrowe, G. J. Smith, M. A. Borton, C. Rey-Sanchez, R. A. Daly, G. Mirfenderesgi, D. W. Hoyt, W. J. Riley, C. S. Miller, G. Bohrer, K. C. Wrighton, Methanogenesis in oxygenated soils is a substantial fraction of wetland methane emissions. *Nat. Commun.* **8**, 1567 (2017).

87. K. E. Miller, C.-T. Lai, E. S. Friedman, L. T. Angenent, D. A. Lipson, Methane suppression by iron and humic acids in soils of the Arctic Coastal Plain. *Soil Biol. Biochem.* **83**, 176–183 (2015).

88. P. Dunfield, R. Knowles, R. Dumont, T. R. Moore, Methane production and consumption in temperate and subarctic peat soils: Response to temperature and pH. *Soil Biol. Biochem.* **25**, 321–326 (1993).

89. J. F. Dean, J. J. Middelburg, T. Röckmann, R. Aerts, L. G. Blauw, M. Egger, M. S. M. Jetten, A. E. E. de Jong, O. H. Meisel, O. Rasigraf, C. P. Slomp, M. H. in't Zandt, A. J. Dolman, Methane feedbacks to the global climate system in a warmer world. *Rev. Geophys.* **56**, 207–250 (2018).

90. K.-Y. Chang, W. J. Riley, S. H. Knox, R. B. Jackson, G. McNicol, B. Poulter, M. Aurela, D. Baldocchi, S. Bansal, G. Bohrer, D. I. Campbell, A. Cescatti, H. Chu, K. B. Delwiche, A. R. Desai, E. Euskirchen, T. Friborg, M. Goeckede, M. Helbig, K. S. Hemes, T. Hirano, H. Iwata, M. Kang, T. Keenan, K. W. Krauss, A. Lohila, I. Mammarella, B. Mitra, A. Miyata, M. B. Nilsson, A. Noormets, W. C. Oechel, D. Papale, M. Peichl, M. L. Reba, J. Rinne, B. R. K. Runkle, Y. Ryu, T. Sachs, K. V. R. Schäfer, H. P. Schmid, N. Shurpali, O. Sonnentag, A. C. I. Tang, M. S. Torn, C. Trotta, E.-S. Tuittila, M. Ueyama, R. Vargas, T. Vesala, L. Windham-Myers, Z. Zhang, D. Zona, Substantial hysteresis in emergent temperature sensitivity of global wetland CH_4 emissions. *Nat. Commun.* **12**, 2266 (2021).

91. S. E. Chadburn, T. Aalto, M. Aurela, D. Baldocchi, C. Biasi, J. Boike, E. J. Burke, E. Comyn-Platt, A. J. Dolman, C. Duran-Rojas, Y. Fan, T. Friborg, Y. Gao, N. Gedney, M. Göckede, G. D. Hayman, D. Holl, G. Hugelius, L. Kutzbach, H. Lee, A. Lohila, F.-J. W. Parmentier, T. Sachs, N. J. Shurpali, S. Westermann, Modeled microbial dynamics explain the apparent temperature sensitivity of wetland methane emissions. *Global Biogeochem. Cy.* **34**, e2020GB006678 (2020).

92. K.-Y. Chang, W. J. Riley, E. L. Brodie, C. K. McCalley, P. M. Crill, R. F. Grant, Methane production pathway regulated proximally by substrate availability and distally by temperature in a high-latitude mire complex. *Eur. J. Vasc. Endovasc. Surg.* **124**, 3057–3074 (2019).

93. A. Findlay, Methane transport in plants. *Nat. Clim. Chang.* **10**, 708 (2020).

94. C. Helfter, M. Gondwe, M. Murray-Hudson, A. Makati, M. F. Lunt, P. I. Palmer, U. Skiba, Phenology is the dominant control of methane emissions in a tropical non-forested wetland. *Nat. Commun.* **13**, 133 (2022).

95. M. J. Carmichael, E. S. Bernhardt, S. L. Bräuer, W. K. Smith, The role of vegetation in methane flux to the atmosphere: Should vegetation be included as a distinct category in the global methane budget? *Biogeochemistry* **119**, 1–24 (2014).

96. N. Horning, J. A. Robinson, E. J. Sterling, W. Turner, S. Spector, *Remote Sensing for Ecology and Conservation: A Handbook of Techniques* (Techniques in Ecology and Conservation Series, Oxford Univ. Press, 2010).

97. A. L. Yagci, L. Di, M. Deng, "The influence of land cover-related changes on the NDVI-based satellite agricultural drought indices," in 2014 IEEE Geoscience and Remote Sensing Symposium (IEEE, Quebec City, 2014), pp. 2054–2057.

98. M. H. Lee, S. B. Lee, Y. D. Eo, M. W. Pyeon, K. I. Moon, S. H. Han, Analysis on the effect of Landsat NDVI by atmospheric correction methods, in *Advances in Civil, Architectural, Structural and Constructional Engineering*, D.-K. Kim, J. Jung, J. Seo, Eds. (CRC, 2016), pp. 375–378.

99. J. W. Rouse, Jr., R. H. Haas, J. A. Schell, D. W. Deering, Monitoring vegetation systems in the Great Plains with ERTS, in *Third Earth Resources Technology Satellite-1 Symposium. Volume I: Technical Presentations,* S. C. Freden, E. P. Mercanti, M. A. Becker, Eds. (SP-351, National Aeronautics and Space Administration, 1974), pp. 309–317.

100. B. R. Deemer, M. A. Holgerson, Drivers of methane flux differ between lakes and reservoirs, complicating global upscaling efforts. *J. Geophys. Res. Biogeosci.* **126**, e2019JG005600 (2021).

101. M. Hayashi, G. van der Kamp, D. O. Rosenberry, Hydrology of prairie wetlands: Understanding the integrated surface-water and groundwater processes. *Wetlands* **36**, 237–254 (2016).

102. H. J. Poffenbarger, B. A. Needelman, J. P. Megonigal, Salinity influence on methane emissions from tidal marshes. *Wetlands* **31**, 831–842 (2011).

103. S. E. Hampton, A. W. E. Galloway, S. M. Powers, T. Ozersky, K. H. Woo, R. D. Batt, S. G. Labou, C. M. O'Reilly, S. Sharma, N. R. Lottig, E. H. Stanley, R. L. North, J. D. Stockwell, R. Adrian, G. A. Weyhenmeyer, L. Arvola, H. M. Baulch, I. Bertani, L. L. Bowman Jr., C. C. Carey, J. Catalan, W. Colom-Montero, L. M. Domine, M. Felip, I. Granados, C. Gries, H.-P. Grossart, J. Haberman, M. Haldna, B. Hayden, S. N. Higgins, J. C. Jolley, K. K. Kahilainen, E. Kaup, M. J. Kehoe, S. MacIntyre, A. W. Mackay, H. L. Mariash, R. M. McKay, B. Nixdorf, P. Nõges, T. Nõges, M. Palmer, D. C. Pierson, D. M. Post, M. J. Pruett, M. Rautio, J. S. Read, S. L. Roberts, J. Rücker, S. Sadro, E. A. Silow, D. E. Smith, R. W. Sterner, G. E. A. Swann, M. A. Timofeyev, *M. Toro*, M. R. Twiss, R. J. Vogt, S. B. Watson, E. J. Whiteford, M. A. Xenopoulos, Ecology under lake ice. *Ecol. Lett.* **20**, 98–111 (2017).

104. C. Krapu, M. Kumar, M. Borsuk, Identifying wetland consolidation using remote sensing in the North Dakota Prairie Pothole Region. *Water Resour. Res.* **54**, 7478–7494 (2018).

105. M. T. Wiltermuth, M. J. Anteau, Is consolidation drainage an indirect mechanism for increased abundance of cattail in northern prairie wetlands? *Wetl. Ecol. Manag.* **24**, 533–544 (2016).

106. J.-F. Pekel, A. Cottam, N. Gorelick, A. S. Belward, High-resolution mapping of global surface water and its long-term changes. *Nature* **540**, 418–422 (2016).

107. USGS, Prairie Pothole Region shapefile (2009); www.sciencebase.gov/catalog/item/54aeaef2e4b0cdd4a5caedf1.

108. ESRI, North America Water Polygons (2021); www.arcgis.com/home/item.html?id=1630b19fafbe4c9589306d967e418088.

109. C. Chatfield, *The Analysis of Time Series: An Introduction* (Texts in Statistical Science Series, CRC, ed. 6, 2003).

110. P. Bloomfield, *Fourier Analysis of Time Series: An Introduction* (Wiley, ed. 2, 2004).

111. E. P. Crist, R. C. Cicone, Comparisons of the dimensionality and features of simulated Landsat-4 MSS and TM data. *Remote Sens. Environ.* **14**, 235–246 (1984).

112. E. P. Crist, R. C. Cicone, A physically-based transformation of Thematic Mapper Data—The TM Tasseled Cap. *IEEE Trans. Geosci. Remote Sens.* **GE-22**, 256–263 (1984).

113. C. Huang, B. Wylie, L. Yang, C. Homer, G. Zylstra, Derivation of a tasselled cap transformation based on Landsat 7 at-satellite reflectance. *Int. J. Remote Sens.* **23**, 1741–1748 (2002).

114. B. Jiang, S.-l. Liang, J.-d. Wang, Z.-q. Xiao, Analysis and prediction of MODIS LAI time series with dynamic harmonic regression model. *J. Remote Sens.* **14**, 13–32 (2010).

115. M. H. A. Baig, L. Zhang, T. Shuai, Q. Tong, Derivation of a tasselled cap transformation based on Landsat 8 at-satellite reflectance. *Remote Sens. Lett.* **5**, 423–431 (2014).

116. G. F. Byrne, P. F. Crapper, K. K. Mayo, Monitoring land-cover change by principal component analysis of multitemporal landsat data. *Remote Sens. Environ.* **10**, 175–184 (1980).

117. C. C. Dymond, D. J. Mladenoff, V. C. Radeloff, Phenological differences in Tasseled Cap indices improve deciduous forest classification. *Remote Sens. Environ.* **80**, 460–472 (2002).

118. R. S. Skakun, M. A. Wulder, S. E. Franklin, Sensitivity of the thematic mapper enhanced wetness difference index to detect mountain pine beetle red-attack damage. *Remote Sens. Environ.* **86**, 433–443 (2003).

119. F. Yuan, K. E. Sawaya, B. C. Loeffelholz, M. E. Bauer, Land cover classification and change analysis of the Twin Cities (Minnesota) Metropolitan Area by multitemporal Landsat remote sensing. *Remote Sens. Environ.* **98**, 317–328 (2005).

120. M. Karlson, M. Ostwald, H. Reese, J. Sanou, B. Tankoano, E. Mattsson, Mapping tree canopy cover and aboveground biomass in Sudano-Sahelian Woodlands using Landsat 8 and Random Forest. *Remote Sens. (Basel)* **7**, 10017–10041 (2015).

121. R Core Team, R: A language and environment for statistical computing, version 4.0.5 (2021); www.R-project.org/.

122. R. J. Hijmans, raster: Geographic data analysis and modeling. R package version 3.4-5 (2020); https://cran.r-project.org/web/packages/raster/index.html.

123. C. Daly, M. Halbleib, J. I. Smith, W. P. Gibson, M. K. Doggett, G. H. Taylor, J. Curtis, P. P. Pasteris, Physiographically sensitive mapping of climatological temperature and precipitation across the conterminous United States. *Int. J. Climatol.* **28**, 2031–2064 (2008).

124. CEC, 2010 Land Cover of North America at 30 meters (2020); www.cec.org/north-american-environmental-atlas/land-cover-2010-landsat-30m/.

125. A. Liaw, M. Wiener, Classification and regression by randomForest. *R news* **2**, 18–22 (2002).

126. M. B. Kursa, W. R. Rudnicki, Feature selection with the Boruta package. *J. Stat. Softw.* **36**, 1–13 (2010).
